# Supplementary material for: Softening of a flat phonon mode in the kagome ScV6Sn6
Source: Nat Commun. 2023 Oct 20;14:6646. doi: 10.1038/s41467-023-42186-6 (PMC10589229; doi:10.1038/s41467-023-42186-6)
Supplement: Supplementary file 1 — Supplementary Information [file 41467_2023_42186_MOESM1_ESM.pdf]

# Supplementary Information

## CONTENTS

SUPPLEMENTARY NOTE 1. Crystal structure 2
A. Crystal structure of CDW phase 2

SUPPLEMENTARY NOTE 2. Characterization of $ScV_6Sn_6$: powder x-ray diffraction and EDX analysis 4

SUPPLEMENTARY NOTE 3. Notes on the crystal structure 4
A. Experimental details 4
B. Crystal structure representations 4

SUPPLEMENTARY NOTE 4. Notes on the phase transition phenomena 6
A. Experimental data 6

SUPPLEMENTARY NOTE 5. Diffuse scattering of $YV_6Sn_6$ 6

SUPPLEMENTARY NOTE 6. Hard x-ray resonant elastic x-ray scattering 7

SUPPLEMENTARY NOTE 7. Inelastic x-ray scattering (IXS) measurements and data analysis 9

SUPPLEMENTARY NOTE 8. Phonon spectrum and flat imaginary phonon modes 9
A. Dynamical matrix 9
B. Low and High-Temperature Phonon spectrum from ab initio Calculation 10
C. Perturbation theory and effective phonon analytic model with only Sn atoms 13
D. Imaginary flat mode 14

SUPPLEMENTARY NOTE 9. Electron-phonon coupling driven imaginary phonon modes 15

SUPPLEMENTARY NOTE 10. Ginzburg Landau theory of CDW phase transition 18

SUPPLEMENTARY NOTE 11. First-principle band structure calculations and ARPES results 18
A. First-principle bulk and surface band structure of $ScV_6Sn_6$ 18
B. ARPES bands 21

References 25

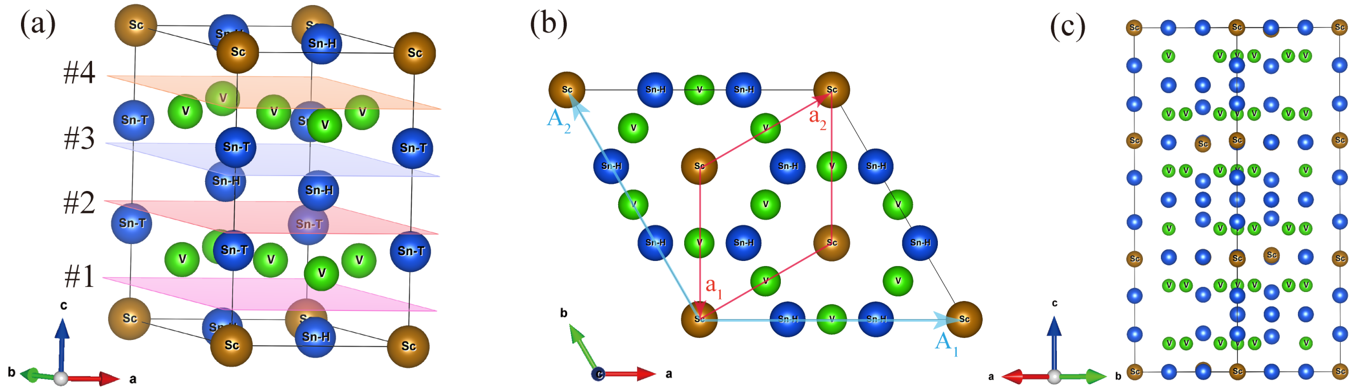


Supplementary Figure 1. (a) The crystal structure $ScV_6Sn_6$ in the pristine phase. Upon cleaving the crystal perpendicularly to the $\hat{\mathbf{z}}$ direction, four possible surface terminations can arise, which are marked with plane #1-#4 in the plot, where the atoms below each plane form the surface. (b) The top view of the conventional cell of the $(\frac{1}{3},\frac{1}{3},\frac{1}{3})$ CDW structure[1], where the blue $A_1, A_2$ denotes the conventional cell basis of the CDW phase, while the red $a_1, a_2$ denotes the pristine cell basis. (c) The side view of the conventional cell of the CDW structure.

## SUPPLEMENTARY NOTE 1. CRYSTAL STRUCTURE

$ScV_6Sn_6$ has a structure in space group (SG) 191 $P6/mmm$ at high temperature, as shown in Supplementary Fig. 1(a). Here, we consider two slightly different crystal structures, the experimental structure measured at 280 $K$ in Ref. [1], and the relaxed structure. Each unit cell contains one Sc atom, six V atoms, and six Sn atoms. The Sc atom forms a triangular lattice for each $xy$ plane. Six V atoms from two layers of Kagome lattice. Two Sn atoms form two layers of trigonal lattices, and the remaining four Sn atoms form two layers of the honeycomb lattice. The comparisons of the experimental structure and the relaxed structure are listed in Supplementary Tab. S1. One marked difference is the relaxation of $Sn^T$ atoms in the unit cell.

The lattice constants measured in this work are $a$=$b$=5.47 Å and $c$=9.17 Å, which is close to the experimental structure in Ref. [1]. The primitive vectors of the unit cell are defined as

$$\boldsymbol{a}_1 = a(1,0,0), \quad \boldsymbol{a}_2 = a(-\frac{1}{2},\frac{\sqrt{3}}{2},0), \quad \boldsymbol{a}_3 = c(0,0,1) \tag{S1}$$

| Structure | Experimental [1] | Relaxed |
|---|---|---|
| $a$ (Å) | 5.4669 | 5.5001 |
| $c$ (Å) | 9.1594 | 9.2150 |
| Sc (1a) | $(0,0,0)$ | $(0,0,0)$ |
| V (6i) | $(\frac{1}{2},0,0.24753)$ | $(\frac{1}{2},0,0.24738)$ |
| $Sn^T$ (2e) | $(0,0,0.67568)$ | $(0,0,0.68245)$ |
| $Sn^{H1}$ (2c) | $(\frac{1}{3},\frac{2}{3},0)$ | $(\frac{1}{3},\frac{2}{3},0)$ |
| $Sn^{H2}$ (2d) | $(\frac{1}{3},\frac{2}{3},\frac{1}{2})$ | $(\frac{1}{3},\frac{2}{3},\frac{1}{2})$ |
| $d_{Sn^T-Sn^T}$ (Å) | 3.218 | 3.363 |

Supplementary Table S1. Comparison of the experimental and relaxed structure of $ScV_6Sn_6$, where $Sn^T$ and $Sn^H$ denote trigonal and honeycomb Sn, respectively. Their Wyckoff positions are also provided. $(i,j,m)$ denotes the position in primitive basis, i.e., $i\boldsymbol{a}_1+j\boldsymbol{a}_2+m\boldsymbol{a}_3$. One marked difference is the relaxation of $Sn^T$ atoms, with the minimum distance between $Sn^T$ atoms presented in the last row.

### A. Crystal structure of CDW phase

We now discuss the crystal structure of the CDW phase. The $(\frac{1}{3},\frac{1}{3},\frac{1}{3})$ CDW results in a $\sqrt{3}\times\sqrt{3}\times 3$ supercell [1]. We compare two slightly different CDW structures. One is from our experimental measurement, which has SG 166 $R\bar{3}m$ with inversion symmetry preserved. The other one is from Ref.[1] measured at 50 $K$, which belongs to SG 155 $R32$ with inversion broken. In Supplementary Tab. S2, we compare the lattice constants and the nearest distance between $Sn^T$ atoms in two CDW structures, which are very close. The nearest distance between two $Sn^T$ atoms in three different layers of the CDW conventional

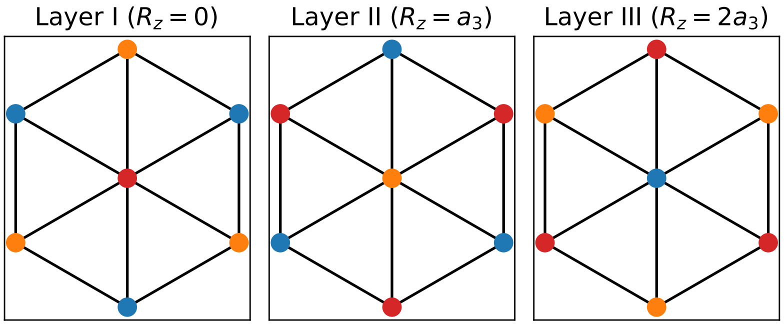


Supplementary Figure 2. Pattern of CDW phase. Red, blue, and orange dots denote three types of non-CDW unit cells. For each layer, the unit cell is tripled (formed by the combination of the red, blue, and orange unit cells). Along $z$ direction, there will also be a $k_z = 2\pi/3$ modulation. Along $z$ direction, the unit cell evolves in red $\rightarrow$ orange $\rightarrow$ blue sequence.

unit cell, where in layers 1 and 3 the distance is shortened while in layer 2 the distance is enlarged compared with the non-CDW structure. The inversion symmetry breaking in structure[1] is due to the small displacements of V and honeycomb Sn atoms. These two CDW structures produce almost identical band structures near $E_f$. A more detailed discussion can be found in Ref.[2]. A top view and a side view of the conventional cell of the CDW phase are shown in Supplementary Fig. 1(b)(c). We

| CDW Structure | Our work | Experimental [1] |
|---|---|---|
| $a$ (Å) | 9.4693 | 9.4561 |
| $c$ (Å) | 27.5153 | 27.4124 |
| $d_{\mathrm{Sn^T-Sn^T}}$, layer 1, 3 (Å) | 3.0924 | 3.073 |
| $d_{\mathrm{Sn^T-Sn^T}}$, layer 2 (Å) | 3.5093 | 3.530 |

Supplementary Table S2. Comparison of the CDW structure of $ScV_6Sn_6$ in our measurement and Ref.[1]. We list the nearest distance between two $Sn^T$ atoms in three different layers of the CDW conventional unit cell, where in layers 1 and 3 the distance is shortened while in layer 2 the distance is enlarged compared with the non-CDW structure.

take the conventional basis $\boldsymbol{A}_i$ of the CDW phase and conventional $\tilde{\boldsymbol{a}}_i$ of the pristine phase (which is a $C_4$ rotation of the basis defined in Eq. S1)

$$\begin{aligned} &\boldsymbol{A}_1 = \tilde{a}(1,0,0),\ \boldsymbol{A}_2 = \tilde{a}(-\frac{1}{2},\frac{\sqrt{3}}{2},0),\ \boldsymbol{A}_3 = \tilde{c}(0,0,1) \\ &\tilde{\boldsymbol{a}}_1 = \tilde{a}(0,-\frac{1}{\sqrt{3}},0),\ \tilde{\boldsymbol{a}}_2 = \tilde{a}(\frac{1}{2},\frac{1}{2\sqrt{3}},0),\ \tilde{\boldsymbol{a}}_3 = \tilde{c}(0,0,\frac{1}{3}) \end{aligned} \tag{S2}$$

where $\tilde{a}, \tilde{c}$ are the CDW lattice constants. We denote the primitive cell basis of the CDW phase as $P_i$, defined as

$$\begin{bmatrix} \boldsymbol{P}_1 \\ \boldsymbol{P}_2 \\ \boldsymbol{P}_3 \end{bmatrix} = \frac{1}{3} \begin{bmatrix} 2 & 1 & 1 \\ -1 & 1 & 1 \\ -1 & -2 & 1 \end{bmatrix} \begin{bmatrix} \boldsymbol{A}_1 \\ \boldsymbol{A}_2 \\ \boldsymbol{A}_3 \end{bmatrix} \tag{S3}$$

We also show the patterns of CDW in Supplementary Fig. 2, where the different colors of dots denote non-equivalent pristine unit cells in the CDW phase. Moving along $z$ direction, the materials will change as ”Layer I” $\rightarrow$ ”Layer II” $\rightarrow$ ”Layer III” and then repeat.

## SUPPLEMENTARY NOTE 2. CHARACTERIZATION OF $SCV_6SN_6$: POWDER X-RAY DIFFRACTION AND EDX ANALYSIS

Supplementary Fig. 3 displays the powder x-ray diffraction data and the LeBail fitting, resulting in lattice parameters , in agreement with the single crystal diffraction data. In figure 3, we present the EDX analysis, showing a ratio of $ScV_{5.97}Sn_{6.07}$.

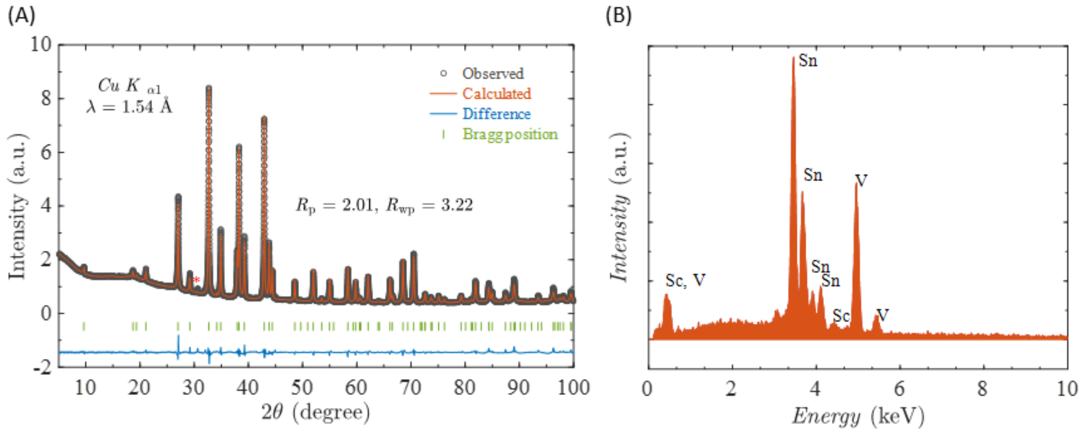


Supplementary Figure 3. **Characterization of $ScV_6Sn_6$.** (A) Powder x-ray diffraction and the LeBail fitting. (B) EDX analysis resulting in composition ratio of Sc:1, V:5.97 and Sn:6.07.

## SUPPLEMENTARY NOTE 3. NOTES ON THE CRYSTAL STRUCTURE

Here we focus on the structural features related to the phase transition phenomena recently observed in the $ScV_6Sn_6$ compound.

### A. Experimental details

Bragg diffraction data were collected at BM01 station, Swiss-Norwegian BeamLines at the European Synchrotron Radiation Facility, Grenoble, France [3]. The wavelength was set to 0.71 Å and data were measured with 0.1 degree slicing, with a single scan per temperature. The temperature was controlled by Cryostream 700+. The data were processed with CrysAlis Pro [4] software, structure was solved with SHEXT [5] and refined with SHELX [6]. Two crystallographic information files (CIFs) are supplemented, for 125K and 80K, as representative for high and low temperature structures.

### B. Crystal structure representations

The layered structure of high temperature (and high symmetry) polymorph is shown in Supplementary Fig. 4. The structure can be presented as sequence of layers with and without Sc. Notably, the Sn-V layers are corrugated with V-Sn-V angle 151.11(8)° (180° would correspond to a flat layer) (Supplementary Fig. 4 top). We found useful to consider the structure as a set of connected coordination polyhedrons. There are only two building blocks, namely triangular prism of Sn atoms centred with V atom, and a bi-caped V-Sn hexagonal prism with Sc atom inside and Sn on the tip of caps (Supplementary Fig. 4 bottom). Those main building units form alternating layers with and without bi-caped hexagonal prisms.

Crystal structure of the low temperature form was solved from single crystal data collected with synchrotron light. The structure is shown at Supplementary Fig. 5. In general, the structure in the low temperature form keeps the same arrangement of the main building units, but structural distortions make them different with corresponding multiplication of the lattice translations. The main deformation relates to displacements of Sc and Sn atoms that results in 3 different bi-caped V-Sn hexagonal prisms. Two prisms show co-axial but not equal displacements of V and Sc atoms as one may see from the inter-atomic distances and angles; according to the direction of deformation with respect to c-axis we mark them as (+), or (-) prisms; those prisms are related by inversion. One more type of prism is symmetric and is not distorted.

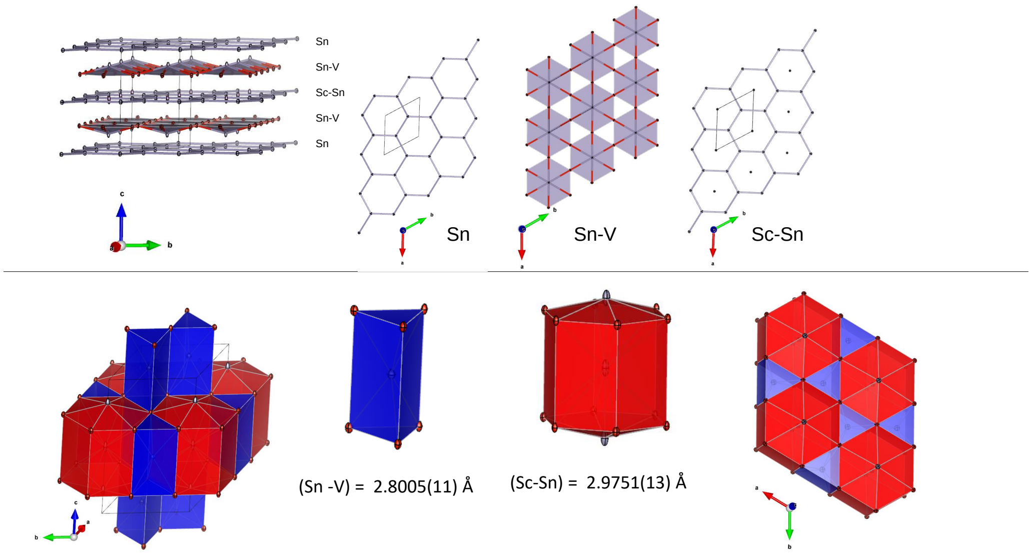


Supplementary Figure 4. Crystal structure of the high symmetry form. Top: representation with a sequence of atomic layers, bottom: representation with main building units.

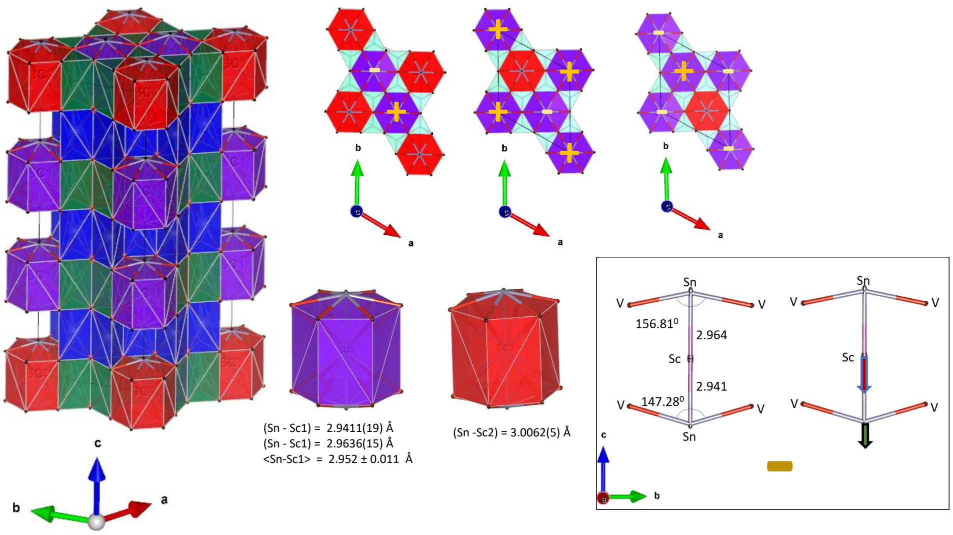


Supplementary Figure 5. Crystal structure of the low temperature form in form of main building units and their layers.

Denoting bi-caped hexagonal prisms according to the deformation pattern we see that the low temperature structure can be rationalized as a 3-layer stacking sequence.

On cooling, we observe a strong diffuse scattering of inelastic nature, indicating a tendency towards a phase transition. As expected for a soft mode transition, the new structure might inherits polarisation (displacement pattern) of the corresponding soft phonon. Here we call this structure ”unstable” or ”missed”, since another structure takes over on cooling. The crystal structure of the hypothetical unstable form can be constructed from the same main building units but with different stacking sequence (Supplementary Fig. 6).

Crystal structure for the low temperature structure was solved in the centrosymmetric space group R-3m. It has to be noted that the lack of inversion center cannot be excluded on a sole basis of diffraction data: R3m structure with 50:50 ratio of inversion twin domains gives the same diffraction pattern as R-3m. R3m structure in particular implies that the adjacent layers (Supplementary Fig. 6) are no longer related by inversion, and the distortions of bi-caped V-Sn hexagonal prism (denoted as (+) and (-)) differ not only by direction but also by the absolute value of atomic displacements. This complication of distortion

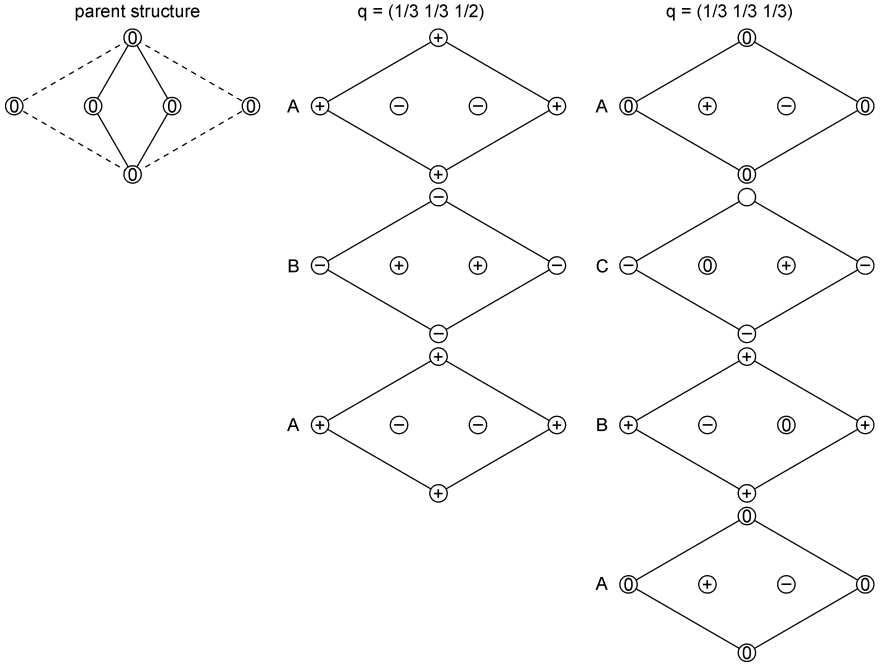


Supplementary Figure 6. Schematics of layers and their stacking in different forms of $ScV_6Sn_6$. Every node in the graph correspond to (distorted) bi-caped V-Sn hexagonal prism, solid lines indicate translations in the (a, b) plane, hexagonal setting. Left: parent structure; middle: "missed" structure associated with soft phonon; right: the low temperature structure.

pattern will not affect the conclusions of a phenomenological analysis of phase transition presented below.

## SUPPLEMENTARY NOTE 4. NOTES ON THE PHASE TRANSITION PHENOMENA

### A. Experimental data

High resolution single crystal diffraction data were collected at ID28 diffraction station at 0.697 Å wavelength with sample-to detector distance of 414 mm and scattering angle up to 130°. Temperature was controlled by Cryostram 700 Plus nitrogen blower and datasets were collected across the transition with 1 K step. Intensity histograms as a function of momentum transfer were traced for the collected peaks; examples are shown in Supplementary Fig. 7, where one can see that close to the basal plane the cell expands upon heating and contracts in axial direction. For the analysis of cell deformation thermal expansion coefficient was evaluated in linear approximation in high temperature phase just above the transition (98-110 K range) and subtracted from the spacing change across the transition (93-100 K). The results are shown as polar plot in Supplementary Fig. 8; angular dependence of deformation across the transition is fitted as $\epsilon_{33}\cos^2(\phi) + \epsilon_{11}\sin^2(\phi)$ with resulting $\epsilon_{11} = 0.9 \cdot 10^{-4}$ and $\epsilon_{33} = -4.4 \cdot 10^{-4}$. Thus, volume jump on heating $\Delta V/V = 2\epsilon_{11} + 2\epsilon_{22} = -2.5 \cdot 10^{-4}$.

Let us summarize the observations. On cooling, we first observe an intense diffuse scattering growing and also relevant phonon mode softening. In general, a pre-transitional behaviour of the lattice susceptibility fits well to mean-field expectations with critical temperature $\mathrm{Tc} \sim 98\,\mathrm{K}$. The expected structural distortion is shown at Supplementary Fig. 6, and is indicated as a "missed" structure. However, at certain temperature before Tc, the system undergoes a first order transition towards different structure (Supplementary Figs. 5,6).

## SUPPLEMENTARY NOTE 5. DIFFUSE SCATTERING OF $YV_6SN_6$

We have carried out diffuse scattering experiments in $YV_6Sn_6$, which has been reported not to show CDW correlations. As shown in Supplementary Fig. 9, no diffuse signal show up in the DS maps, in agreement with [1].

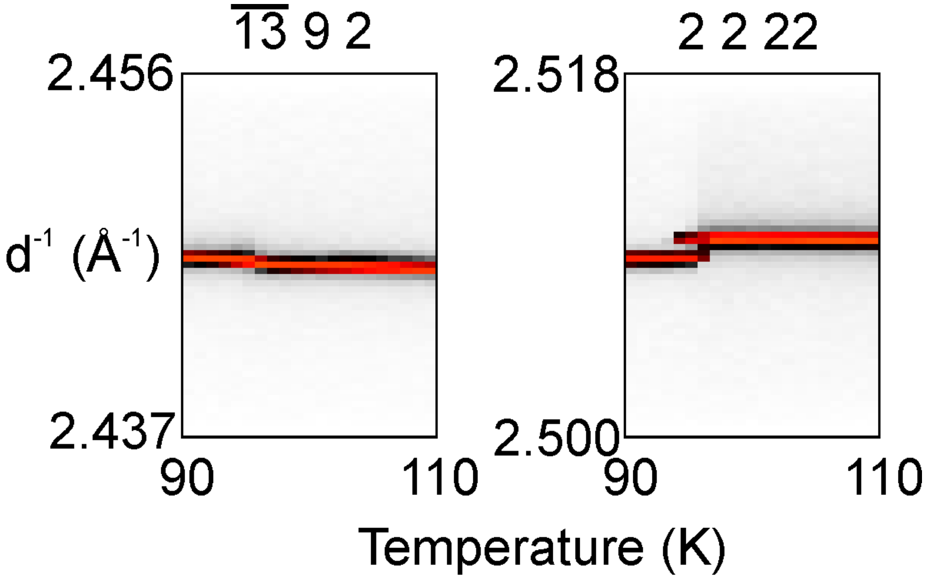


Supplementary Figure 7. Selected intensity histograms as a function of temperature and momentum transfer across the phase transition in $ScV_6Sn_6$.

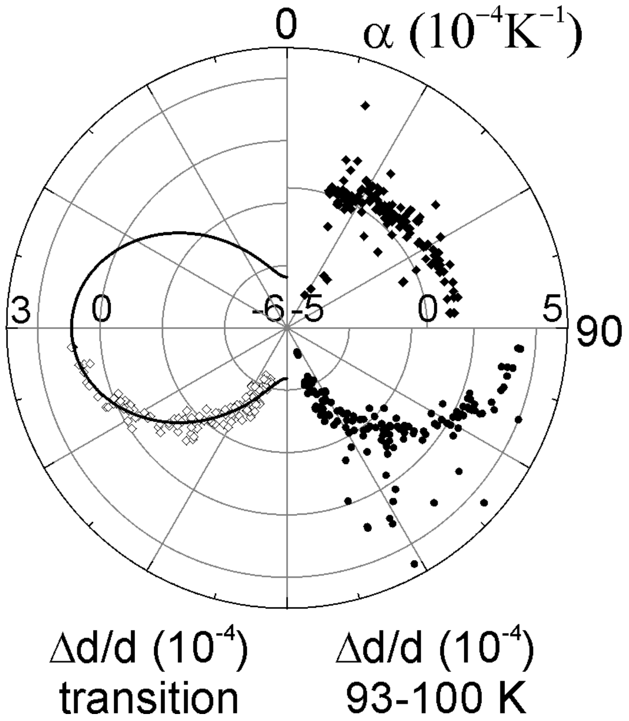


Supplementary Figure 8. Polar plot representation of thermal expansion of $ScV_6Sn_6$ (upper right quadrant) and its lattice deformation across the phase transition – lower right quadrant for raw data and lower left quadrant with subtracted thermal expansion contribution and the removal of the outliers. Corresponding fit is given as solid line in the left part. Polar axis is along lattice vector *c*.

## SUPPLEMENTARY NOTE 6. HARD X-RAY RESONANT ELASTIC X-RAY SCATTERING

Resonant x-ray spectroscopies are described by the virtual processes that promote a core electron to some empty higher energy level. In a resonant hard x-ray diffraction (RXS) experiment, a high energy photon that is absorbed by a core electron, and subsequently decays with re-emission of a photon, is coherent throughout the crystal, thus giving the usual Bragg diffraction condition. At the resonance, the scattering form factor is expressed as

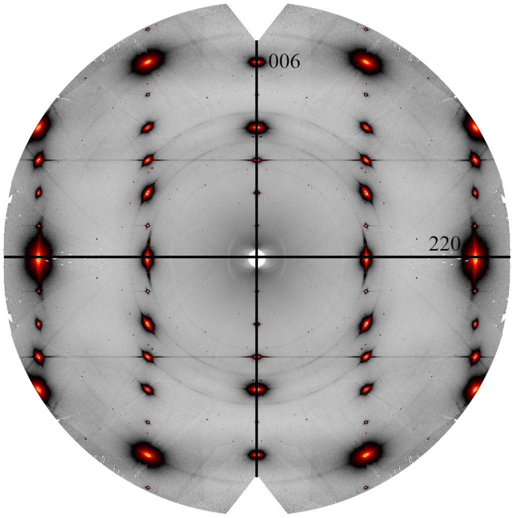


Supplementary Figure 9. **Diffuse scattering of $YV_6Sn_6$**. Temperature dependence of the (h h l) plane showing no diffuse precursors indicative of soft modes.

$$F = \sum_a e^{i\mathbf{Q}\cdot\mathbf{R_a}} \left(f_{0a} + f'_a + i f''_a\right) \tag{S4}$$

where $\mathbf{R}_a$ stands for the position of the scattering ion $a$, $\mathbf{Q}$ is the diffraction vector, and $f_0$ is the Thomson scattering factor due to the scattering of a free charged particle. The resonant part, *f'*+ *if"*, is called the *anomalous* atomic scattering factor, and is given by

$$f' + if'' = -m_e\omega^2 \sum_n \int_{E_F}^{\infty} \frac{M_{ng}^{o*} M_{ng}^{i}}{E_n - E_g - \hbar\omega - i\frac{\Gamma(E)}{2}} dE \tag{S5}$$

Here, $\hbar\omega$ is the photon energy, $m_e$ the electron mass, $E_g$ the ground state energy, $E_n$ and $\Gamma$ are the energy and inverse lifetime of the excited states. $M_{ng}$, the transition matrix elements of the dipole operator. At resonance, $E_n$- $E_g$$\approx$ $\hbar\omega$.

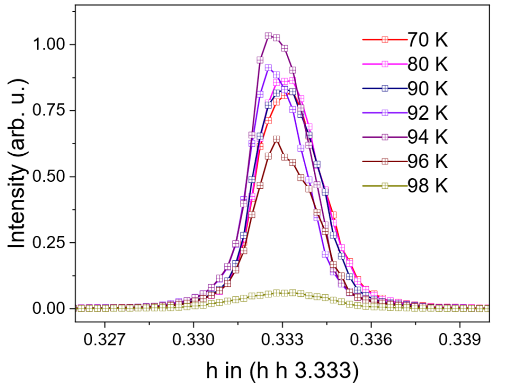


Supplementary Figure 10. Temperature dependence of the CDW reflection measured with incident energy $E_i = 5.462\,\mathrm{keV}$.

The resonant hard x-ray diffraction (RXS) data was taken by setting the incident energy ($E_i$) to the absorption K-edge of vanadium, i.e. $E_i$= 5.462 keV, which corresponds to the $1s \rightarrow 4p$ dipole allowed the transition of vanadium. The sample was glued with the $c$-axis in the scattering plane. Supplementary Fig. 10 displays the temperature dependence of the $\frac{1}{3}\frac{1}{3}\frac{10}{3}$ CDW peak and fitted to Lorentzian profiles. The intensity and linewidth are plotted in Supplementary Fig. 1(I) of the main text.

## SUPPLEMENTARY NOTE 7. INELASTIC X-RAY SCATTERING (IXS) MEASUREMENTS AND DATA ANALYSIS

In the following Supplementary Figs. 11-14, we detail the inelastic x-ray scattering (IXS) measurements as a function of temperature and momentum, taken at European Synchrotron Research Facility (ESRF, ID28 beamline) with energy resolution, $\Delta E = 3\,\mathrm{meV}$ (incident beam energy was 17.8 keV and the horizontally scattered beam was analyzed by a silicon analyzer Si (9, 9, 9)) and at Argonne Photon Source (APS, HERIX beamline) with energy resolution $\Delta E = 1.5\,\mathrm{meV}$ (incident beam energy was 23.72 keV and the horizontally scattered beam was analyzed by silicon analyzer, Si (12, 12, 12).

Inelastic x-ray scattering measures the dynamic structure factor, $S(Q,\omega)$, that is proportional to the imaginary part of the phonon dynamic susceptibility $\chi''(\mathbf{Q},\omega)$ ($\chi''(\mathbf{Q},\omega) = S(\mathbf{Q},\omega)\cdot\left(1-e^{-\omega/K_BT}\right)$).

For an anharmonic phonon the dynamic structure factor $S(Q,\,\omega)$ is given by [7]:

$$S(Q,\omega) = \frac{[n(\omega)+1]Z(Q)\,4\omega^{\Gamma_q/\pi}}{[(\omega-\omega_q)^2+\Gamma_q^2][(\omega+\omega_q^2)]+\Gamma_q^2} \tag{S6}$$

where $Z(Q) = \exp((-2W_Q)\,|\boldsymbol{Q}\cdot e|^2/2M$, with the exponential being the Debye-Waller factor ($\exp((-2W_Q) = \langle\exp((i\mathbf{G}\cdot\mathbf{u}_q)\rangle^2$, where $\mathbf{G}$ is a reciprocal lattice vector and $\mathbf{u}_q$ is the displacement of the $q$-th atom), $e$ is the polarization vector, $M$ is the mass of the atom, and $\Gamma_q$ and $\omega_q$ are the phonon linewidth and frequency, respectively.

The IXS scans were fitted to damped harmonic oscillators (DHO) for phonons convoluted with the experimental resolution, detailed in the supplementary information of [8].

The damping ratio is obtained from $\Gamma/\tilde{\omega}_q$, where $\tilde{\omega}_q$ is the frequency of the phonon renormalized by the real part of the susceptibility $\omega_q = \sqrt{\tilde{\omega}_q^2 - \Gamma_q^2}$ [9].

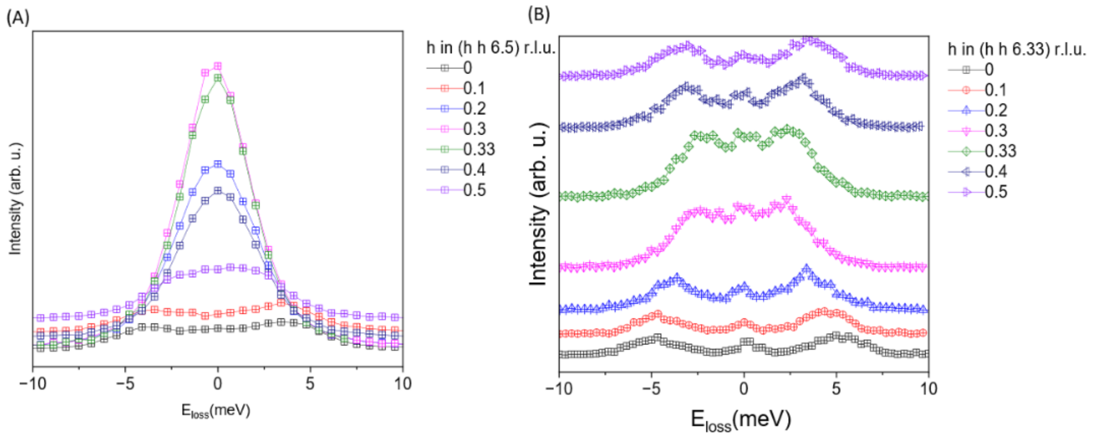


Supplementary Figure 11. **IXS spectra at 150 K.** (A) Momentum dependence of the low energy longitudinal mode for l=6.5 ($\Delta E = 3\,\mathrm{meV}$, ESRF). (B) Momentum dependence of the low energy longitudinal mode for l=6.33 ($\Delta E = 1.5\,\mathrm{meV}$, APS). The phonon softening is visible around h h=0.3 r.l.u.

## SUPPLEMENTARY NOTE 8. PHONON SPECTRUM AND FLAT IMAGINARY PHONON MODES

In this section, we describe the theoretically calculated phonon spectrum. This is an abbreviated version of a full theoretical description, to appear in Ref. [2].

### A. Dynamical matrix

We use $u_{i\mu}(\mathbf{R},t)$ to describe the $\mu$-direction displacement of atom $i$ at time $t$ and position $\mathbf{R}+\mathbf{r}_i$, where $\mathbf{R}$ denote the position vector of unit-cell and $r_i$ denote the location of $i$-th atom in the unit cell. The dynamics of atom displacement are described by eh following equation of motion

$$M_i\partial_t^2 u_{i\mu}(\mathbf{R},t) = \sum_{\mathbf{R}',\nu,j}\Phi_{i\mu,j\nu}(\mathbf{R}'-\mathbf{R})u_{j\nu}(\mathbf{R},t) \tag{S7}$$

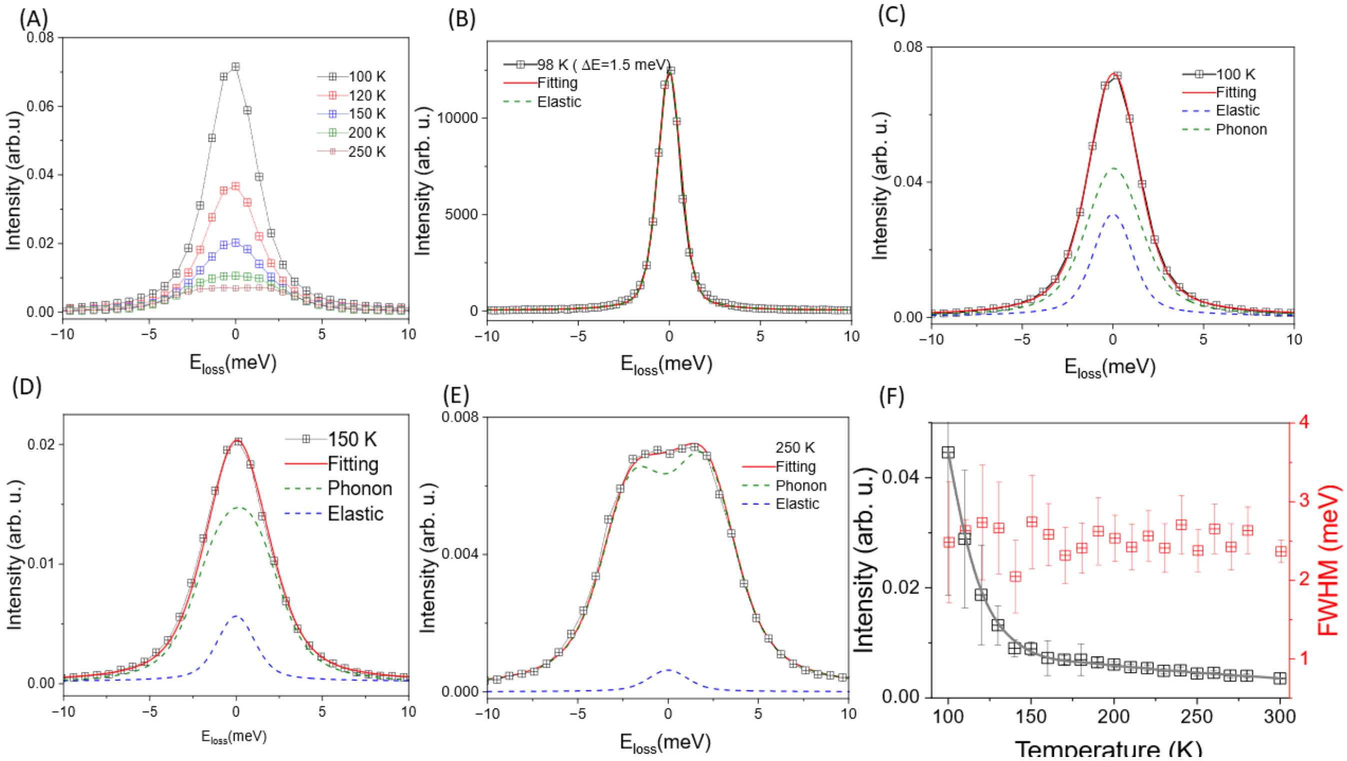


Supplementary Figure 12. **Analysis of the soft mode at l=6.5**. (A) Temperature dependence of the 1/3 1/3 6.5 phonon. (B) Fitting details of the IXS spectra at 98 K ($\Delta E = 1.5\,\mathrm{meV}$, APS). (C-E) Fitting details at 100, 150 and 250 K ($\Delta E = 3\,\mathrm{meV}$, ESRF). Above 250 K, taking into account the energy resolution and the phonon damping, the IXS scans consist on broad Stokes and anti-Stokes phonons. Below 250 K, the elastic central peak (CP) develops. (F) Temperature dependence of the phonon intensity and its linewidth. The phonon linewidth is constant with T.

where $\Phi_{i\mu,j\nu}(\mathbf{R}-\mathbf{R}')$ are the matrices of force constants and $M_i$ is the mass of $i$-th atom. The solutions of Eq. S7 can be written as

$$u_{i\mu}(\mathbf{R},t) = \frac{1}{\sqrt{N}}\sum_{\mathbf{k}}\frac{1}{\sqrt{M_i}}\eta_n^{i\mu}(\mathbf{k})e^{i\mathbf{k}(\mathbf{R}+\mathbf{r}_i)}e^{-i\omega_{n,\mathbf{k}}t} \tag{S8}$$

where $N$ is the total number of unit cells, and $\mathbf{k}$ is the momentum of the phonon mode. The eigenvector $\eta_n^{i\mu}(\mathbf{k})$ and frequency and $\omega_{n,\mathbf{k}}$ that characterize the $i$-th vibration mode can be obtained by solving

$$\omega_{n,\mathbf{k}}^2 v_n^{i\mu}(\mathbf{k}) = \sum_{j\nu} D_{i\mu,j\nu}(\mathbf{k}) v_n^{j\nu}(\mathbf{k}) \tag{S9}$$

with $D_{i\mu,j\nu}(\mathbf{k}) = \frac{1}{\sqrt{M_i}}\Phi_{i\mu,j\nu}(\mathbf{k})\frac{1}{\sqrt{M_j}}$ the dynamical matrix.

### B. Low and High-Temperature Phonon spectrum from ab initio Calculation

We numerically obtain the phonon spectrum by calculating the force constant matrix via density functional theory (DFT) as implemented in Vienna *ab-initio* Simulation Package (VASP) [10–12] and Quantum Espresso (QE) [13]. The generalized gradient approximation (GGA) with Perbew-Burke-Ernzerhof (PBE) scheme [14] is adopted for the exchange-correlation functional. The structure was fully relaxed before the phonon calculation with a force convergence criteria of $10^{-3}$ eV/Å. The phonon spectra and force constants by VASP are extracted via PHONOPY code with density functional perturbation theory (DFPT) [15], using a $3 \times 3 \times 2$ supercell with a Γ-centered $3 \times 3 \times 2$ $k$-mesh. The energy cutoff is 320 eV. For calculations in QE, we adopted the standard solid-state pseudopotentials (SSSP) [16], and the kinetic-energy cutoff for wavefunctions is set to be 90 Ry. For the self-consistent calculations, a $12 \times 12 \times 6$ Monkhorst-Pack k-mesh was implemented with an energy convergence

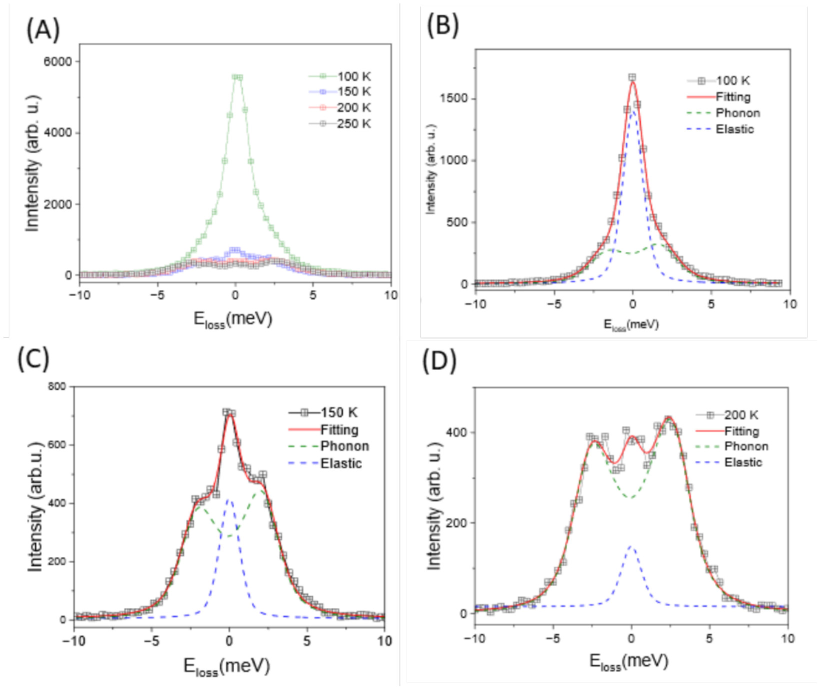


Supplementary Figure 13. Analysis of the soft mode at l=6.33 ($\Delta E = 1.5\,\mathrm{meV}$, APS). (A) Temperature dependence of the raw IXS spectra. (B-D) Fitting details, as in Supplementary Fig. 11. As shown in the main text, the frequency of the mode remains finite at the $T_{\mathrm{CDW}}$.

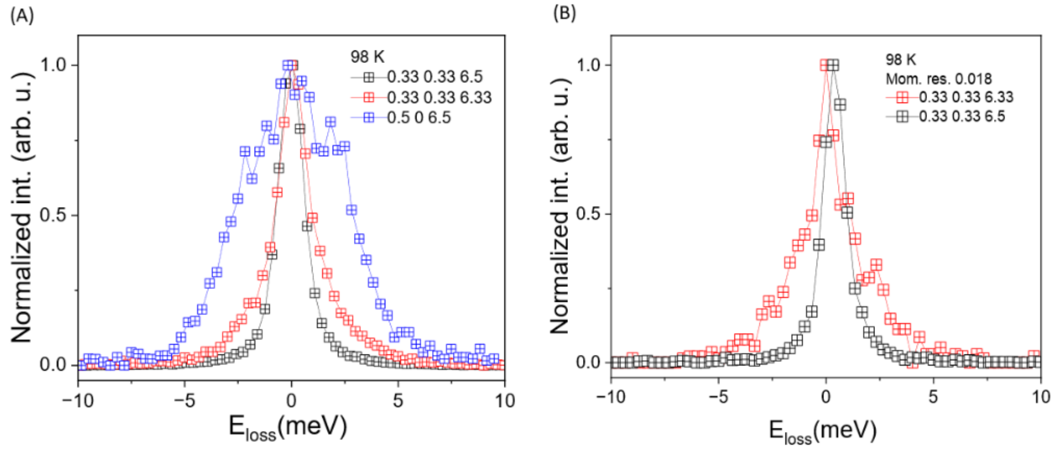


Supplementary Figure 14. IXS scans at 98 K ($\Delta E = 1.5\,\mathrm{meV}$, APS). (A) Comparison of the IXS spectra at three different momenta. (B) Comparison of the IXS spectra for l=1/2 and l=1/3 with momentum resolution of $0.018\,\mathrm{A}^{-1}$.

criteria of $10^{-8}$ Ry. For electron-phonon coupling (EPC) calculations, a denser $24 \times 24 \times 12$ $k$-mesh is employed with an energy convergence criteria of $10^{-13}$ Ry. Spin-orbit coupling is not included in the structural relaxation and phonon calculations.

From the phonon spectrum of $ScV_6Sn_6$ in Supplementary Fig. 16 (a), one can identify the instability in the $k_z = \pi$ plane, which contains the most negative squared frequency (the reason for this will be explained analytically later (SUPPLEMENTARY NOTE 9; full description and calculation can be found in Ref. [2].), and is related to positive string constant between two trigonal Sn atoms induced by the electron-phonon coupling). The atom projection reveals that the dominant contribution is the out-of-plane vibration of triangular Sn atoms and Sc atoms, as plotted in Supplementary Fig. 17.

To understand better the evolution of the phonon spectrum, we perform calculations at different temperatures, as shown in Supplementary Fig. 18. The temperature-dependent phonon spectra are calculated via a smearing method within the harmonic approximation. We however employ the stable, positive, phonon spectrum at high temperatures to later analytically compute the field theory correction to the phonon frequency, and show the renormalization of imaginary frequency, which gives a strong analytic agreement with the low-temperature phonon spectrum [2].

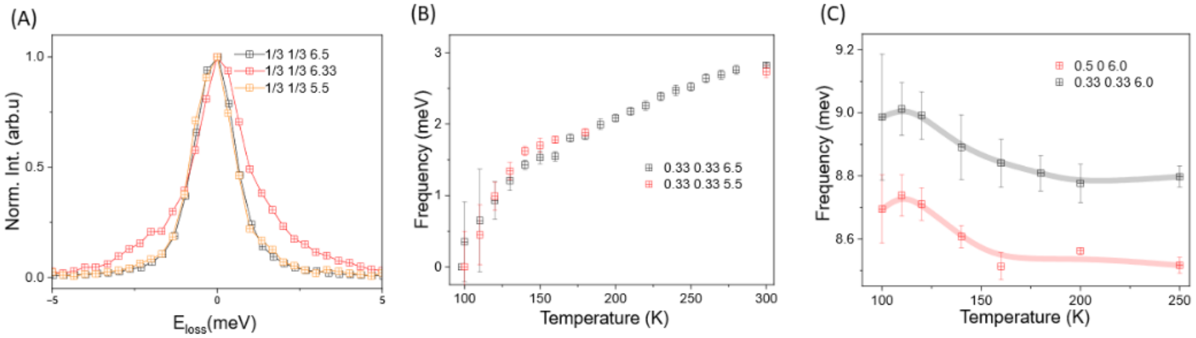


Supplementary Figure 15. **More phonons**. ($\Delta E = 1.5\,\mathrm{meV}$, APS). (A) Comparison of the 1/3 1/3 5.5 spectrum at 98 K. Similar to l=6.5, the l=5.5 mode consists of a resolution-limited elastic line. (B) Temperature dependence of the l=5.5 mode. (C) Temperature dependence of the purely acoustic branches 0.5 0 6.0 (*M* point) and 0.33 0.33 6.0 (*K* point), showing a phonon hardening down to 100 K.

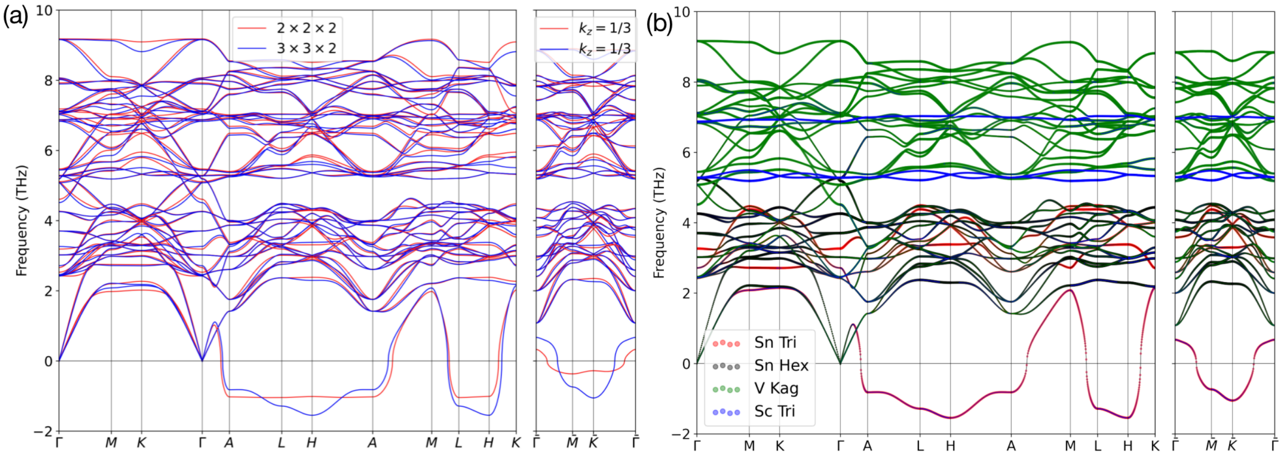


Supplementary Figure 16. Phonon spectrum of $ScV_6Sn_6$ by VASP. (a) The blue and red lines denote the results calculated with $3 \times 3 \times 2$ and $2 \times 2 \times 2$ supercells. The path at $k_z = 1/3$ plane is plotted on the right side. (b) The phonon spectrum is projected according to the site projection, including the trigonal Sn (red), hexagonal Sn (black), kagome V (green), and trigonal Sc (blue). The imaginary phonon band in the $k_z = \pi$ plane is mostly contributed by $Sn^T$ atoms. If we further resolve the modes to vibrations in $x$, $y$, and $z$ directions, we can see the instability is caused by the out-of-plane vibration of $Sn^T$ atoms.

In Supplementary Fig. 19, we also plot the phonon spectrum weighted by the magnitude of $\lambda_{\mathbf{q}\nu}$, which characterizes the strength of electron-phonon coupling by integrating $k$-mesh over the whole Brillouin zone. $\lambda_{\mathbf{q}\nu}$ is given by

$$\lambda_{\mathbf{q}\nu} = \frac{\gamma_{\mathbf{q}\nu}}{\pi\hbar N(\varepsilon_{\mathrm{F}})\omega_{\mathbf{q}\nu}}, \tag{S10}$$

where $N(\varepsilon_{\mathrm{F}})$ is the density of states at Fermi surface, $\omega_{\mathbf{q}\nu}$ is the phonon frequency. $\gamma_{\mathbf{q}\nu}$ is the linewidth or inverse lifetime of a phonon mode, given by

$$\begin{aligned} \gamma_{\mathbf{q}\nu} &= 2\pi\omega_{\mathbf{q}\nu}\sum_{i,j}\int\frac{d^3k}{\Omega}|g_{\mathbf{k},\mathbf{q}}^{ij,\nu}|^2\delta(\varepsilon_{\mathbf{q},i}-\varepsilon_{\mathrm{F}})\delta(\varepsilon_{\mathbf{k}+\mathbf{q},j}-\varepsilon_{\mathrm{F}}) \\ g_{\mathbf{k},\mathbf{q}}^{ij,\nu} &= \left(\frac{\hbar}{2M\omega_{\mathbf{q}\nu}}\right)^{\frac{1}{2}}\langle\psi_{\mathbf{k},i}|\frac{dV_{\mathrm{SCF}}}{du_{\mathbf{q}\nu}}\eta_{\mathbf{q}\nu}|\psi_{\mathbf{k}+\mathbf{q},j}\rangle. \end{aligned} \tag{S11}$$

where $g_{\mathbf{k},\mathbf{q}}^{ij,\nu}$ is the EPC matrix element. Here, $\psi_{k,i}$ is the electronic wave function at momentum k, of the band $i$, $V_{\mathrm{SCF}}$ is the Kohn-Sham potential, $u$ is the atomic displacement, and $\eta_{\mathbf{q}\nu}$ is the phonon eigenvector. From Supplementary Fig. 19, we observe a strong normalization of the low-branch phonon mode at $k_z = \pi$ plane. In SUPPLEMENTARY NOTE 9, we will analyze how the electron correction drives the instability in the phonon modes.

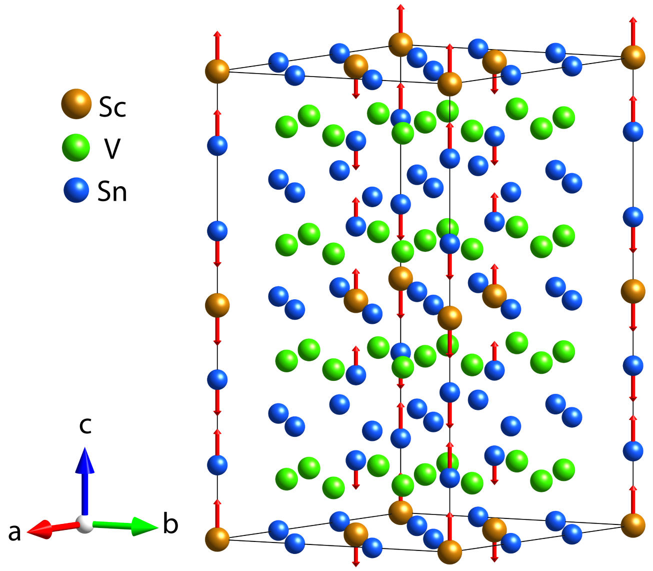


Supplementary Figure 17. Vibration mode corresponding to $q = (\frac{1}{3}, \frac{1}{3}, \frac{1}{2})$, which is mainly contributed by the out-of-plane vibration of $\mathrm{Sn}^{\mathrm{T}}$ atoms and Sc atoms.

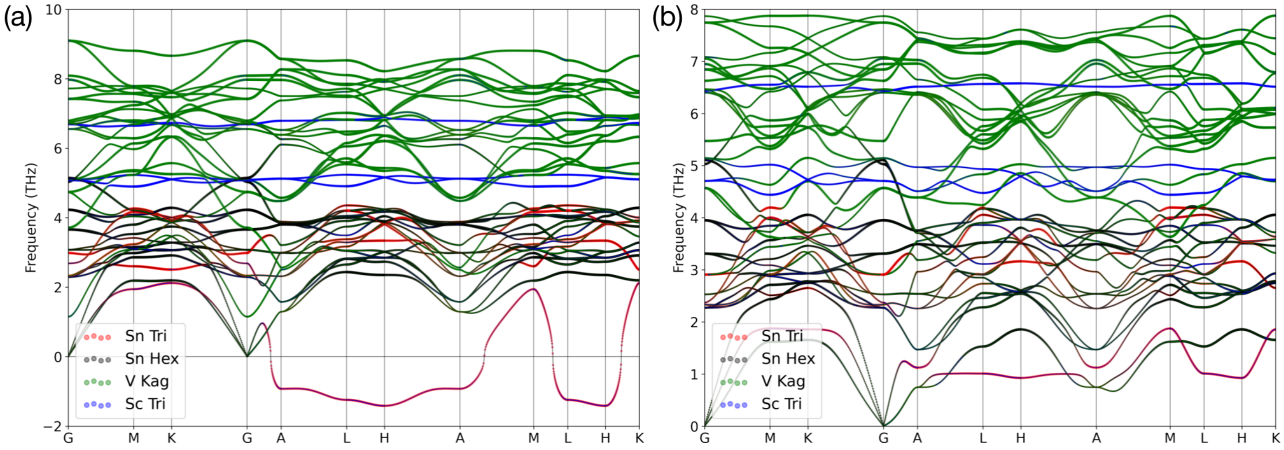


Supplementary Figure 18. Phonon spectrum of $\mathrm{ScV_6Sn_6}$ by QE with a $3 \times 3 \times 2$ $q$-mesh, weighted by atomic projection with Fermi-Dirac smearing values of $0.014$ eV and $0.4$ eV to simulate (a) low-T and (b) high-T phonon, respectively.

### C. Perturbation theory and effective phonon analytic model with only Sn atoms

In this section, we take the force-constant matrix from DFT calculation and derive an analytical phonon model with only Sn atoms via perturbation theory. The full details of the derivation is shown in Ref. [2]. The masses of three types of atoms are

$$M_{Sn} = 118.71u, \quad M_{Sc} = 44.96u, \quad M_V = 50.94u. \tag{S12}$$

where Sn is the heaviest one and the low-energy phonon spectrum is formed by this heaviest atom (Supplementary Fig. 16).

We use perturbation theory and derive the effective dynamical matrix $D^{eff}$ with only Sn atoms

$$D^{eff}(\mathbf{k}) = \frac{1}{M_{Sn}}\Phi^{Sn-Sn} - \frac{1}{M_{Sn}}\left[\frac{1}{\sqrt{M_V}}\Phi^{Sn-V} \quad \frac{1}{\sqrt{M_{Sc}}}\Phi^{Sn-Sc}\right]\begin{bmatrix}\frac{1}{M_V}[\Phi^{V-V}] & \frac{1}{\sqrt{M_V M_{Sn}}}[\Phi^{V-Sc}] \\ \frac{1}{\sqrt{M_{Sc} M_V}}[\Phi^{Sc-V}] & \frac{1}{M_{Sc}}[\Phi^{Sc-Sc}]\end{bmatrix}\begin{bmatrix}\frac{1}{\sqrt{M_V}}\Phi^{V-Sn} \\ \frac{1}{\sqrt{M_{Sc}}}\Phi^{Sc-Sn}\end{bmatrix} \tag{S13}$$

We use $\Phi^{A-B}$ to represent the force constant matrix between different sets of atoms. By only keeping the nearest-neighbor

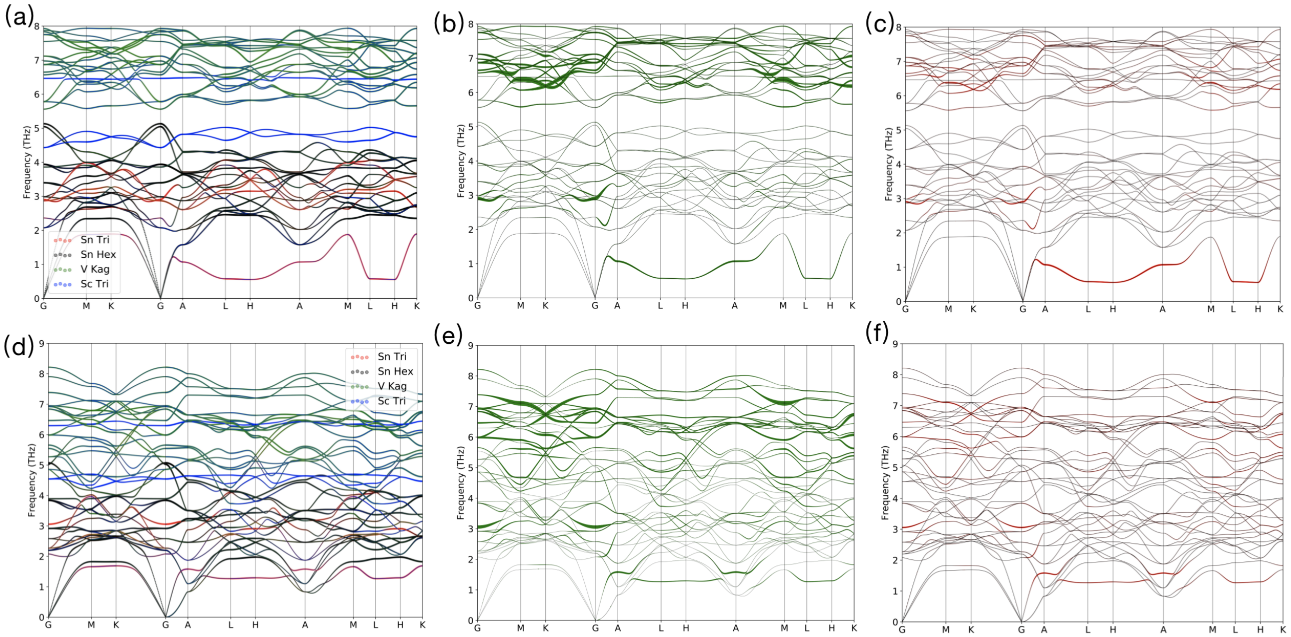


Supplementary Figure 19. Phonon spectrum calculated by QE with a $2 \times 2 \times 2$ $q$-mesh, weighted by atomic projection (left), phonon linewidth (middle) and electron-phonon coupling (right) with Fermi-Dirac smearing values of $0.4$ eV (top) and $0.8$ eV (bottom). Evidently, the previously unstable mode has a relatively large contribution to EPC, which overlaps with the projection of $\mathrm{Sn^T}$.

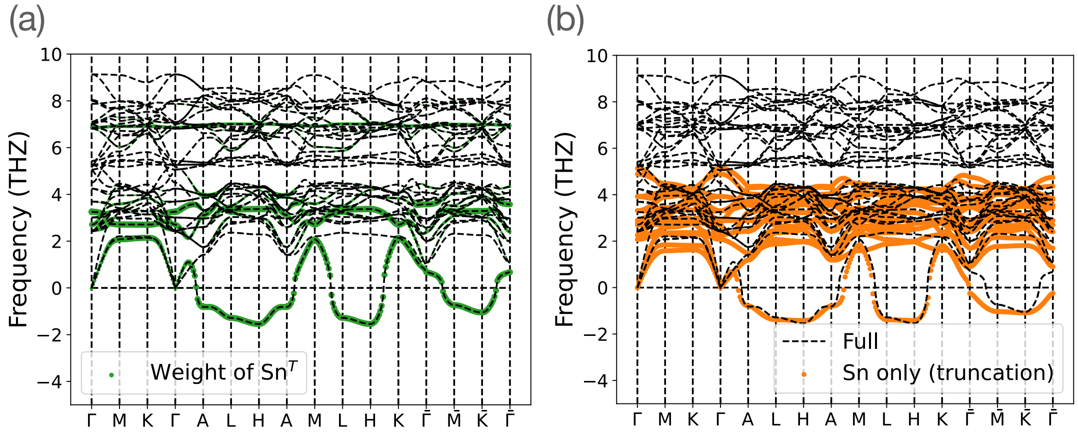


Supplementary Figure 20. (a) Phonon spectrum of the full model, where the green dots mark the weights of the $z$ direction movement of trigonal Sn modes. (b) Comparison between the spectrum of the full model and the spectrum of the effective model with only Sn atoms and truncated coupling (to the nearest neighbor). Here $\bar{\Gamma} = (0, 0, 0\frac{1}{3})$, $\bar{M} = (\frac{1}{2}, 0, \frac{1}{3})$, $\bar{K} = (\frac{1}{3}, \frac{1}{3}, \frac{1}{3})$.

contribution in the effective force constant matrix, we can very well reproduce the phonon spectrum of the full model as shown in Supplementary Fig. 20.

### D. Imaginary flat mode

Based on the effective phonon model with only Sn atoms, we now analyze the properties of the imaginary phonon model. The full details of the derivation is shown in Ref. [2] The imaginary phonon mode is mostly formed by the out-of-plane vibration of

trigonal $\text{Sn}^T$ as shown in Supplementary Fig. 20 (a). We then observe this imaginary mode is relatively flat in the $k_z = \pi$ plane. From our effective model, we find the $z$ direction displacement of $\text{Sn}^T$ will strongly couple to the $z$ direction displacement of the other $\text{Sn}^T$ with the same $x, y$ coordinates. By only keeping this dominant coupling, we build an effective one-dimensional (1D) model to describe the vibration of two trigonal Sn atoms

$$D_{1D}(\mathbf{k}) = \begin{bmatrix} d_1 + d_2 & -d_1 e^{ik_z \Delta r_z} - d_2 e^{ik_z(-1+\Delta r_z)} \\ -d_1 e^{-ik_z \Delta r_z} - d_2 e^{ik_z(1-\Delta r_z)} & d_1 + d_2 \end{bmatrix} \tag{S14}$$

$d_1 = -2.61\text{THz}^2$ represents the coupling in the dynamical matrix between two trigonal Sn of the same unit cell. $d_2 = 4.06\text{THz}^2$ represents the coupling in the dynamical matrix between two trigonal Sn of the nearby unit cell along $z$ direction. $\Delta r_z$ is the distance between two Sn atoms within the same unit cell. It is more convenient to work on the even-odd basis

$$\eta^{e,z}(\mathbf{R}) = \frac{1}{\sqrt{2}}[\eta^{1z}(\mathbf{R}) - \eta^{2z}(\mathbf{R})], \quad \eta^{a,z}(\mathbf{R}) = \frac{1}{\sqrt{2}}[\eta^{1z}(\mathbf{R}) + \eta^{2z}(\mathbf{R})] \tag{S15}$$

In the new basis, the dynamical matrix becomes

$$\tilde{D}_{1D}(\mathbf{k}) = \begin{bmatrix} 2d_1 + d_2 + d_2\cos(k_z) & -id_2\sin(k_z) \\ id_2\sin(k_z) & d_2 - d_2\cos(k_z) \end{bmatrix} \tag{S16}$$

where the first and second row/column denotes $\eta^{e,z}$ and $\eta^{a,z}$ respectively. The phonon frequency is

$$\omega_1(\mathbf{k})^2 = d_1 + d_2 - \sqrt{d_1^2 + d_2^2 + 2d_1 d_2\cos(k_z)}, \quad \omega_2(\mathbf{k})^2 = d_1 + d_2 + \sqrt{d_1^2 + d_2^2 + 2d_1 d_2\cos(k_z)} \tag{S17}$$

Since $d_1 < 0, d_2 > 0$, $\omega_1(\mathbf{k})^2$ develops a minimum at $k_z = \pi$, with $\omega_1(k_z = \pi)^2 = 2d_1 < 0$. This is the imaginary phonon mode that we observed in the phonon spectrum. The corresponding eigenvector in the real space is

$$\eta^{1z}(\mathbf{R}) = -\eta_0(-1)^{i_z}, \quad \eta^{2z}(\mathbf{R}) = \eta_0(-1)^{i_z} \tag{S18}$$

where we use $i_z$ to denote $i_z$-th unit cell along $z$ directions and $\eta_0$ is a real number, that matches the vibration mode of each one-dimensional chain in Supplementary Fig. 17.

## SUPPLEMENTARY NOTE 9. ELECTRON-PHONON COUPLING DRIVEN IMAGINARY PHONON MODES

In this section, we demonstrate the imaginary phonon is induced by the electron-phonon coupling. We first introduce the electron-phonon model. The Hamiltonian reads

$$\begin{aligned} &H = H_c + H_{ph} + H_g \\ &H_c = \sum_{\mathbf{k},\alpha\gamma,\sigma} t_{\alpha\gamma}(\mathbf{k}) c^\dagger_{\mathbf{k},\alpha\sigma} c_{\mathbf{k},\gamma\sigma} \\ &H_{ph} = \sum_{\mathbf{R},i,\mu} \frac{(P_{i\mu}(\mathbf{R}))^2}{2M_\alpha} + \sum_{\mathbf{R}',\mathbf{R}'i\mu,j\nu} \frac{1}{2}\Phi_{i\mu,j\nu}(\mathbf{R}-\mathbf{R}') u_{i\mu}(\mathbf{R}) u_{j\nu}(\mathbf{R}') \\ &H_g = \sum_{\mathbf{q},\mathbf{k},\alpha,\gamma,\delta,\sigma} \frac{g^{\alpha\gamma i\mu}_{\mathbf{k},\mathbf{q}}}{\sqrt{N}} u_{i\mu}(\mathbf{q}) c^\dagger_{\mathbf{k}+\mathbf{q},\alpha\sigma} c_{\mathbf{k},\gamma\sigma} \end{aligned} \tag{S19}$$

$c^\dagger_{\mathbf{k},\alpha\sigma}$ creates an electron with momentum $\mathbf{k}$, orbital $\alpha$ and spin $\sigma$. $t_{\alpha\gamma}(\mathbf{k})$ is the hopping matrix of electrons in the momentum space. $u_{i\mu}(\mathbf{R})$ denotes the $\mu$ direction displacement field of $i$-th atom and $P_{i\mu}(\mathbf{R})$ denotes the corresponding momentum of displacement field. $g^{\alpha\gamma,i\mu}_{\mathbf{k},\mathbf{q}}$ is the tensor that characterize the electron-phonon couplings. $N$ is the total number of unit cells.

It is more convenient to consider the band basis of electrons. We introduce the electron operator on the band basis ($\gamma_{\mathbf{k},n\sigma}$), which also allows us to write the electron-phonon coupling in the band basis $h^{nm,i\mu}_{\mathbf{k},\mathbf{q}}$ where $n, m$ are the band indices. By integrating our $\gamma$ electrons, we derive an effective theory of phonon fields using perturbation theory where the electron-phonon coupling is treated perturbatively. We then find the following correction to the force constant matrix [2]

$$\Phi^{corr}_{i\mu,i'\nu}(\mathbf{q}) = -\sum_{n,m,\sigma,\mathbf{k}} \frac{h^{nm,i'\nu}_{\mathbf{k},\mathbf{q}} h^{mn,i\mu}_{\mathbf{k}+\mathbf{q},-\mathbf{q}}}{N} \frac{-n_F(\epsilon_{\mathbf{k}+\mathbf{q},n}) + n_F(\epsilon_{\mathbf{k},m})}{\epsilon_{\mathbf{k}+\mathbf{q},n} - \epsilon_{\mathbf{k},m}} \tag{S20}$$

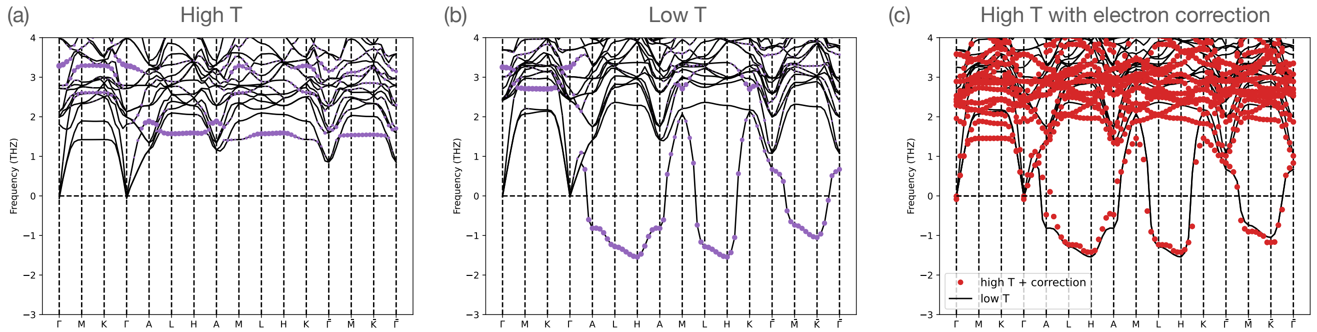


Supplementary Figure 21. (a) Phonon spectrum from DFT calculation at high temperature $T$ =1.2eV. (B) Phonon spectrum from DFT calculation at low-temperature (zero temperature). (C) Comparison between phonon spectrum derived by adding electron correction to the high-temperature phonon spectrum (red dots) and low-temperature phonon spectrum derived from DFT. In (a), (b), the purple dots mark the weight of the $z$-direction mirror-even vibration mode of the trigonal Sn atoms ($\frac{1}{\sqrt{2}}(u_{Sn_1^T z}(\mathbf{R}) - u_{Sn_2^T z}(\mathbf{R}))$).

Combining the corrections induced by electrons with the original force constant matrix, we obtain the following effective force constant matrix

$$\Phi^{eff}_{i\mu,i'\nu}(\mathbf{q}) = \Phi_{i\mu,i'\nu}(\mathbf{q}) - \sum_{n,m,\sigma,\mathbf{k}} \frac{h^{nm,i'\nu}_{\mathbf{k},\mathbf{q}} h^{mn,i\mu}_{\mathbf{k+q},-\mathbf{q}}}{N} \frac{-n_F(\epsilon_{\mathbf{k+q},n}) + n_F(\epsilon_{\mathbf{k},m})}{\epsilon_{\mathbf{k+q},n} - \epsilon_{\mathbf{k},m}}. \tag{S21}$$

We use Eq. S20 to investigate the effect of electron-phonon coupling. We first describe the construction of the model (Eq. S19). The hopping matrix $t_{\alpha\gamma}(\mathbf{k})$ and the non-interacting force constant matrix $\Phi_{\alpha\mu,\gamma\nu}(\mathbf{q})$ are given by the DFT calculations in the pristine phase. Here the non-interacting force constant matrix refers to the force constant matrix calculated at high temperature ($T = 1.2$eV) where the electron correction is suppressed due to the thermal effect (which can be seen from Eq. S21). $g^{\alpha\gamma,i\mu}_{\mathbf{k},\mathbf{q}}$ is derived from the Gaussian approximation introduced in Ref. [17]. We focus on the correction to the vibration of the heaviest atom of the system, Sn, and keep the contribution from $p$ orbitals of Sn atoms which strongly couples to the vibration of Sn atoms. We also truncate the electronic bands by only taking electronic bands in the energy window $[E_F - 20\text{meV}, E_f + 20\text{meV}]$.

We first discuss the high-temperature (non-interacting) phonon model whose spectrum is shown in Supplementary Fig. 21 (a). We observe that low-branch phonon modes are mostly formed by the $z$-driection movements of trigonal Sn atoms and are extremely flat. This is because the coupling between phonon modes along the direction, that is perpendicular to the vibration direction, is usually weak. Here the low-brunch phonon has $z$-direction vibration and then the $xy$-direction coupling is weaker which leads to a weak in-plane dispersion and flat mode. The flatness of the phonon bands indicates the low-energy branch phonon mode can be described by an effective 1D chain model as introduced in the effective one-dimensional model introduced in Eq. S14 but with different parameters in the high-temperature phase ($d_1 = 0.67\text{THz}^2, d_2 = 3.22\text{THz}^2$). We can see both $d_1, d_2$ are positive which indicates the absence of imaginary phonon (from Eq. S17).

We next describe the effect of electron-phonon interaction. In Supplementary Fig. 21, we show both the high-temperature (non-interacting) phonon spectrum with electron correction and the low-temperature phonon spectrum derived from DFT. We observe a good match between the two methods, where a relatively flat imaginary band with leading order instability at H has been found in both methods.

We are now in the position to discuss the origin of imaginary mode. We find that the dominant electron-phonon coupling from Gaussian approximation [17] takes the form of (see Ref. [2])

$$H_g = \tilde{g} \sum_{\mathbf{R},\sigma} u_{e,z}(\mathbf{R}) c^\dagger_{\mathbf{R},e,\sigma} c_{\mathbf{R},e,\sigma} \tag{S22}$$

where $\tilde{g} = 2.23\mathring{A}^{-1}u^{-1}eV$ we have introduced the following electron field $c_{\mathbf{R},e,\sigma}$ and phonon field $u_{\mathbf{R},ez}$ where both are even under mirror $z$ transformation

$$c_{\mathbf{R},e,\sigma} = \frac{1}{\sqrt{2}}\left(c_{\mathbf{R},(Sn_1^T,p_z),\sigma} - c_{\mathbf{R},(Sn_2^T,p_z),\sigma}\right), \quad u_{ez}(\mathbf{R}) = \frac{1}{\sqrt{2}}(u_{Sn_1^T,z}(\mathbf{R}) - u_{Sn_2^T,z}(\mathbf{R})), \quad u_{oz}(\mathbf{R}) = \frac{1}{\sqrt{2}} \tag{S23}$$

The coupling in Eq. S22 produces a correction term ($H^{corr}$) to the phonon Hamiltonian (see Ref. [2]) which is induced by the charge fluctuations of mirror-even electron $c_{\mathbf{R},e,\sigma}$ which is characterized by the corresponding charge susceptibility $\chi_e$ of $c_{\mathbf{R},e,\sigma}$ electron The momentum-dependency of the static susceptibility $\chi_e(\mathbf{q}, i\Omega_n = 0)$ is shown in the main text Fig. 4,

where the peak near H and $\bar{\text{K}}$ comes from the weak nesting of the Fermi surface as illustrated in Supplementary Fig. 24. We observe that $\chi_e(\mathbf{q}, i\Omega_n = 0)$ curve is similar to the dispersion of the tight-binding model on a triangular lattice. In addition, the Wannier centers of $c_{\mathbf{R},e,\sigma}$ locates at $\mathbf{r} = \frac{1}{2}\boldsymbol{a}_3$ and indeed form a triangular lattice. These motivate us to perform a transform $\chi_e(\mathbf{q}, i\Omega_n = 0)$ to the real-space and find

$$\chi_e(\mathbf{R}, i\Omega_n = 0) \approx \chi^{on\text{-}site}\delta_{\mathbf{R},\mathbf{0}} + \sum_{i=1,...,6} \chi^{xy}\delta_{\mathbf{R},\mathbf{R}_i^{xy}} + \sum_{i=1,2} \chi^{z}\delta_{\mathbf{R},\mathbf{R}_i^{z}} \tag{S24}$$

which consists of a strong on-site term $\chi^{on\text{-}site} = 0.289$, and a relatively weak nearest-neighbor in-plane term ($\chi^{xy} = -0.009$) and a relatively weak nearest-neighbor out-of-plane term ($\chi^{z} = -0.016$). $\mathbf{R}_i^{xy}$ and $\mathbf{R}_i^{z}$ characterize the in-pane and out-of-pane nearest-neighrbor sites respectively. We note that, due to the weak nesting, we do not observe any sharp peak in the $\chi_e(\mathbf{q}, i\Omega_n = 0)$, which indicates a moderate momentum dependency of $\chi_e(\mathbf{q}, i\Omega_n = 0)$ (Fig. 4 of main text). Consequently, transforming to the real space, the susceptibility only has a sizeable value in a short distance. This is in contrast to the case where $\chi_e(\mathbf{q}, i\Omega_n = 0)$ shows a strong peak at certain momentum $\mathbf{Q}$ which happens if there is a strong nesting in the Fermi surface. In the extreme case with $\chi_e(\mathbf{q}, i\Omega_n = 0) = \tilde{\chi_e}\delta_{\mathbf{q},\mathbf{Q}}$, the corresponding susceptibility in the real-space behaves like $\chi_e(\mathbf{R}, i\Omega_n = 0) = \tilde{\chi_e}e^{i\mathbf{Q}\cdot\mathbf{R}}$ which indicates a long-range correlation in the real space with a non-decaying susceptibility and $\mathbf{Q}$ modulation. In another limit without any momentum dependency $\chi_e(\mathbf{q}, i\Omega_n = 0) = \tilde{\chi_e}$, the corresponding susceptibility in the real-space behaves like $\chi_e(\mathbf{R}, i\Omega_n = 0) = \tilde{\chi_e}\delta_{\mathbf{R},\mathbf{0}}$ which indicates a strong on-site fluctuations. Our current susceptibility is in between two limiting cases, which gives a short-range correlation with dominant on-site and weak nearest-neighbor terms.

From Eq. S16 and Eq. S24, we reach the following effective dynamical matrix [2]

$$D^{eff,eo}(\mathbf{q}) = \tilde{D}_{1D}(\mathbf{q}) + \begin{bmatrix} d_3(\mathbf{q}) & 0 \\ 0 & 0 \end{bmatrix} = + \begin{bmatrix} 2d_1 + d_2 + d_2\cos(q_z) + d_3(\mathbf{q}) & -id_2\sin(q_z) \\ id_2\sin(q_z) & d_2 - d_2\cos(q_z) \end{bmatrix} \tag{S25}$$

where

$$d_3(\mathbf{q}) = -\frac{\tilde{g}^2}{M_{Sn}}\chi_e(\mathbf{q}, i\Omega_n = 0) = -\frac{\tilde{g}^2}{M_{Sn}}\left(\chi^{on\text{-}site} + 2\chi^{xy}\left(\cos(q_1) + \cos(q_2) + \cos(q_1 + q_2)\right) + 2\chi^z\cos(q_z)\right) \tag{S26}$$

with $q_1 = \mathbf{q}\cdot\boldsymbol{a}_1, q_2 = \mathbf{q}\cdot\boldsymbol{a}_2$.

A direct diagonalization leads to the following spectrum

$$\omega^2_{1/2,\mathbf{q}} = d_1 + d_2 + \frac{d_3(\mathbf{q})}{2} \pm \sqrt{d_2\sin(q_z)^2 + \left(d_1 + d_2\cos(q_z) + \frac{d_3(\mathbf{q})}{2}\right)^2} \tag{S27}$$

We note that $\chi^{on\text{-}site}$ merely introduce a normalization, which motivates us to define

$$\begin{aligned} \tilde{d}_1 &= d_1 + \frac{-\tilde{g}^2}{2M_{Sn}}\chi^{on\text{-}site}, \\ d_{xyz}(\mathbf{q}) &= d_{xy}\left(\cos(q_1) + \cos(q_2) + \cos(q_1 + q_2)\right) + d_z\cos(q_z), \quad d_{xy} = -\frac{\tilde{g}^2}{M_{Sn}}\chi_{xy}, \quad d_z = -\frac{\tilde{g}^2}{M_{Sn}}\chi_z \end{aligned} \tag{S28}$$

Due to the electron corrections the renormalized $\tilde{d}_1 = -0.807\text{THz}^2$ becomes negative. $d_{xy} = -0.081\text{THz}^2, d_z = -0.046\text{THz}^2$ are relatively weak, which allows us to perform an expansion in powers of $d_{xyz}(\mathbf{q})$ and only keep the zeroth order and leading-order term

$$\omega^2_{1/2,\mathbf{q}} \approx \left(\tilde{d}_1 + d_2 \pm \sqrt{d_2\sin(q_z)^2 + \left(\tilde{d}_1 + d_2\cos(q_z)\right)^2}\right) + d_{xyz}(\mathbf{q}) \tag{S29}$$

where the first term in the big bracket is the same dispersion as the effective one-dimensional model but with parameters $\tilde{d}_1, d_2$, as we discussed near Eq. S17, the minimum of this term locates at $q_z = \pi$. At $q_z = \pi$ plane, we realize

$$\begin{aligned} \omega_{1,\mathbf{q}}\Big|_{q_z=\pi} &= 2\tilde{d}_1 + \frac{d_{xyz}(\mathbf{q})}{2} + d_z(\mathbf{q}) \\ \omega_{2,\mathbf{q}}\Big|_{q_z=\pi} &= 2d_2 + d_{xyz}(\mathbf{q}) \end{aligned} \tag{S30}$$

From $\tilde{d}_1 < 0$, we find imaginary modes at the whole $k_z = \pi$ plane. The weak $d_{xyz}(\mathbf{q})$ term will then introduces a weakly in-plane dispersion that selects the H point to be the leading order instability. Note that $d_{xyz}(\mathbf{q})$ also introduce additional $q_z$ dependency. However, since $|d_z|$ is much smaller than $\tilde{d}_1, d_2$, such correction will not affect the fact that the most negative mode locates at $q_z = \pi$.

## SUPPLEMENTARY NOTE 10. GINZBURG LANDAU THEORY OF CDW PHASE TRANSITION

We next analyze the CDW transition based on the Ginzburg Landau theory. Two sets of order parameters are considered. The first set of the order parameters describing the soft phonon mode at H= $(\frac{1}{3}\frac{1}{3}\frac{1}{2})$ and -H=$(\frac{-1}{3}\frac{-1}{3}\frac{-1}{2})$

$$\phi \sim u_{soft}(\mathbf{q} = \frac{1}{3}\frac{1}{3}\frac{1}{2}), \quad \phi^\dagger \sim u_{soft}(\mathbf{q} = \frac{-1}{3}\frac{-1}{3}\frac{-1}{2}) \tag{S31}$$

where $\phi$ is the corresponding order parameter and $u_{soft}(\mathbf{q})$ denotes the phonon fields at $\mathbf{q}$ with lowest energy. From our DFT and perturbation calculation, the phonon field $u_{soft}(\mathbf{q} = H)$ with the lowest energy at H forms an H3 irreducible representation of the little group at H.

The second set of the order parameters describing the CDW phase with wavevectors $\bar{\mathrm{K}} = \frac{1}{3}\frac{1}{3}\frac{1}{3}$. Note that there are four non-equivalent but symmetry related $\bar{\mathrm{K}}$ points. Our order parameters $\psi_1, \psi_2$ are taken to be

$$\psi_1 \sim u_{soft}(\mathbf{q} = \frac{1}{3}\frac{1}{3}\frac{1}{3}), \quad \psi_1^\dagger \sim u_{soft}(\mathbf{q} = \frac{-1}{3}\frac{-1}{3}\frac{-1}{3}), \quad \psi_2 \sim u_{soft}(\mathbf{q} = \frac{-1}{3}\frac{-1}{3}\frac{1}{3}), \quad \psi_2^\dagger \sim u_{soft}(\mathbf{q} = \frac{1}{3}\frac{1}{3}\frac{-1}{3}) \tag{S32}$$

where $u_{soft}(\bar{\mathrm{K}})$ forms a P1 irreducible representation of the little group at $\bar{\mathrm{K}}$.

We next construct the Ginzburg Landau theory of the order parameters $\phi, \psi_1, \psi_2$. The symmetry allowed Ginzburg Landau free energy (up to quadratic term) consists of three terms, the free energy of $\phi$ fields $L_\phi$, the free energy of $\psi_1, \psi_2$ fields $L_\psi$, and the term that couples $\phi$ with $\psi_1, \psi_2$.

$$L = L_\phi + L_\psi + L_{\phi\psi} \tag{S33}$$

where each term takes the form of [2]

$$\begin{aligned} L_\phi &= m^\phi |\phi|^2 + u^\phi |\phi|^4 \\ L_\psi &= m^\psi (|\psi_1|^2 + |\psi_2|^2) + \frac{\gamma}{2}(\psi_1^3 + \psi_1^{\dagger,3} + \psi_2^3 + \psi_2^{\dagger,3}) + u^{\psi,1}(|\psi_1|^4 + |\psi_2|^4) + u^{\psi,2}|\psi_1|^2|\psi_2|^2 \\ L_{\phi\psi} &= u^{\phi\psi}|\phi|^2(|\psi_1|^2 + |\psi_2|^2) \end{aligned} \tag{S34}$$

$m^\phi, m^\psi$ can be understood as the excitation gap of the soft phonon at H and $\bar{\mathrm{K}}$ respectively. $\gamma$ is the coefficient of the cubic term of $\psi$ fields. $u^\phi, u^{\psi,1}, u^{\psi,2}, u^{\phi\psi}$ are real numbers that characterize the quadratic term of order parameters. We can start from the decoupled limit with $u^{\phi\psi} = 0$. Then $L_\phi$ (H point order) describes a second-order phase transition, while $L_\psi$ ($\bar{\mathrm{K}}$ point order) describes a first-order phase transition due to the cubic term in $L_\psi$. These are consistent with experimental observations where the collapsing of phonons (at H) follows the behaviors of second-order transition, while the CDW transition (at $\bar{\mathrm{K}}$) is first-order. In addition, because the CDW transition with wavevector $\bar{\mathrm{K}}$ is first-order, it does not require the collapsing of $\bar{\mathrm{K}}$ phonon (equivalently speaking, it does not require $m^\psi$ goes to zero at transition temperature).

We next consider a non-zero $u^{\phi\psi}$ and explain why the collapsing of phonon at H could induce a CDW transition at $\bar{\mathrm{K}}$, where a detailed analysis is given in Ref. [2]. When we reduce the temperature from the high-temperature pristine phase to the low-energy CDW phase, the excitation energy of phonon with momentum H gradually reduces, which is equivalent to the reduction of $m^\phi$. The reduction of the excitation energy combining with the flat nature of the phonon mode near H indicates a strong fluctuation of $\phi$ fields. Since $|\phi|^2$ couples to $|\psi_1|^2 + |\psi_2|^2$ via $L_{\phi\psi}$, after integrating out the fluctuation of $\phi$ fields, the values of $u^{\psi,1}, u^{\psi,2}$ will be renormalized and reduced. Such normalization effect becomes more and more dominant as we decrease the temperature (reduce $m^\phi$) which will finally drive a first-order transition to the CDW phase by the condensation of the $\psi_1, \psi_2$ fields.

## SUPPLEMENTARY NOTE 11. FIRST-PRINCIPLE BAND STRUCTURE CALCULATIONS AND ARPES RESULTS

In this appendix, we present extended results on the electronic band structure of $ScV_6Sn_6$. We start by computing the *ab-initio* bulk band structure of $ScV_6Sn_6$, in both the pristine and charge-density wave (CDW) phases[1]. Additionally, we also analyze the four different surface terminations of $ScV_6Sn_6$ arising when the crystal is cleaved perpendicularly to the $\hat{\mathbf{z}}$ direction[18–21] and compute the corresponding surface states in the pristine phase. Moreover, we present additional ARPES data along various high-symmetry lines of the Brillouin zone and compare it with the *ab-initio* bulk and surface band structure obtained previously.

### A. First-principle bulk and surface band structure of $ScV_6Sn_6$

Due to the short penetration length of photons in crystals, the photoemission signal measured in ARPES experiments is not only dependent on the bulk electronic band structure, but also on the electronic surface states of the crystal. In this section, we present *ab-intio* results on the bulk and surface bands structure of $ScV_6Sn_6$.

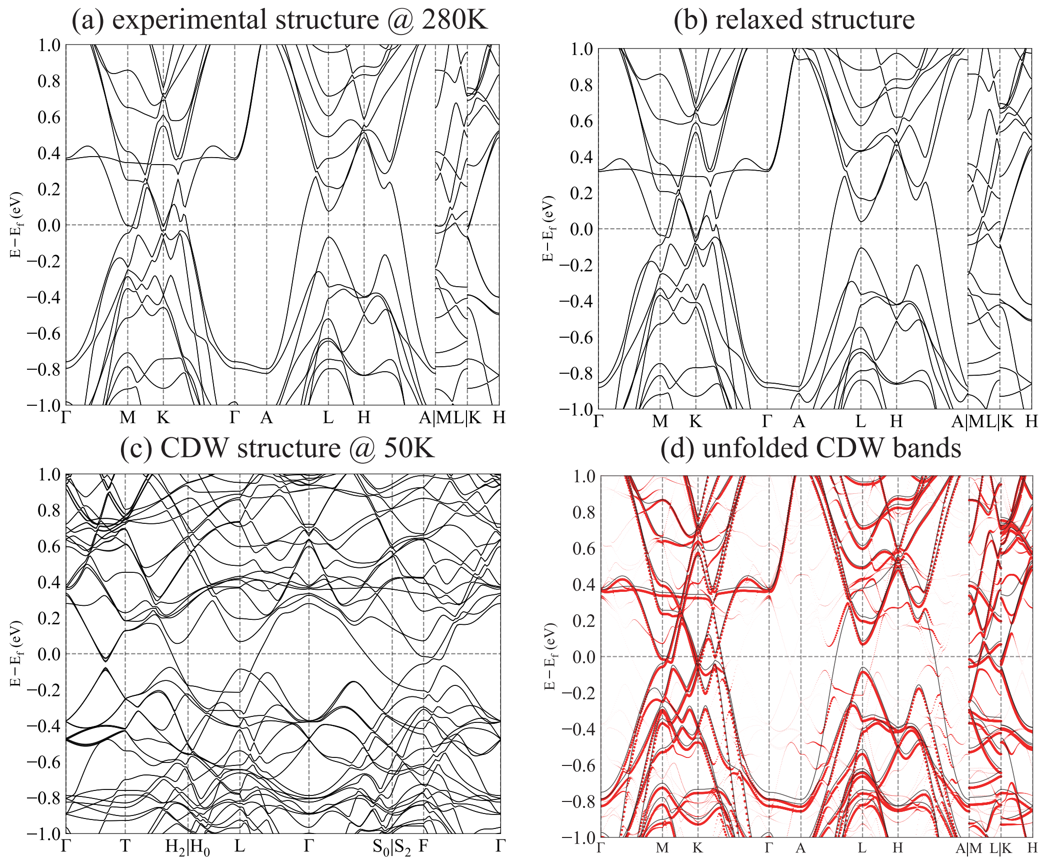


Supplementary Figure 22. The band structure of $ScV_6Sn_6$ in the pristine and $(\frac{1}{3}, \frac{1}{3}, \frac{1}{3})$ CDW phase. (a)(b) The band structure with SOC of the experimental[1] and relaxed pristine structure, respectively. The Fermi energy of the relaxed structure is about 30 meV higher on the $k_z = 0$ plane. (c) The band structure of the CDW phase in the BZ of the primitive cell in SG 155. (d) Unfolded band structure of the CDW phase to the pristine BZ of SG 191, where the red dots are unfolded bands while the grey lines are bands of the experimental pristine structure.

| Orbitals | Sc, $d$ | V, $d$ | $Sn^T$, $p_z$ | $Sn^T$, $p_{x,y}$ | $Sn^H$, $p$ |
|---|---|---|---|---|---|
| DOS@$E_f$ (Relaxed) | 0.19 | 2.94 | 0.39 | 0.05 | 0.14 |
| DOS@$E_f$ (Experimental [1]) | 0.13 | 2.87 | 0.35 | 0.05 | 0.10 |

Supplementary Table S3. Density of states (DOS) of different orbitals at the Fermi energy $E_F$.

We calculate the electron band structure for both the relaxed structure and the experimental structure in the pristine phase (Supplementary Fig. 22(a)(b)) with SOC, where the Fermi energy $E_f$ of the relaxed structure on $k_z = 0$ plane is about 30 meV higher than the experimental one. The orbital projections of the experimental structure are shown in Supplementary Fig. 23. The band that crosses $E_f$ along $A$-$L$ and $H$-$A$ and forms part of the large Fermi surface is mainly $d_{xz}, d_{yz}$ of V and $p_z$ of trigonal Sn. The density of the states of different orbitals at Fermi energy are given in Supplementary Tab. S3. We can observe only $d$ orbitals of V and $p_z$ orbitals of trigonal Sn are the relevant low-energy degrees of freedom. We also investigate the possible Fermi surface nesting, where the Fermi surface is given in Supplementary Fig. 24(a)(c). In Supplementary Fig. 24(b)(d), we mark two nesting vectors with one near H point and the other one near $\bar{K} = \frac{1}{3}\frac{1}{3}\frac{1}{3}$. The CDW band structure with SOC in the primitive Brillouin zone is provided in Supplementary Fig. 22(c). In Supplementary Fig. 22(d), the band structure in the CDW phase has been "unfolded" back to the Brillouin zone of the pristine phase[22–24]. We observe a gap opening due to the formation of CDW order along $A$-$L$ and $H$-$A$ lines.

In preparation for ARPES experiments, the $ScV_6Sn_6$ crystals are usually cleaved perpendicularly to the $\hat{\mathbf{z}}$ direction[18–21] Within the pristine phase, this results in four possible surface terminations that have been marked with #1-#4 in Supplementary Fig. 1(a). In Supplementary Fig. 25, we illustrate the surface band structures of $ScV_6Sn_6$ in the pristine phase, arising in the four possible surface terminations #1-#4. The band structures from Supplementary Fig. 25 are computed directly using VASP within a slab geometry of four layers. In order to compute all four surface terminations in Supplementary Fig. 1(a), we consider

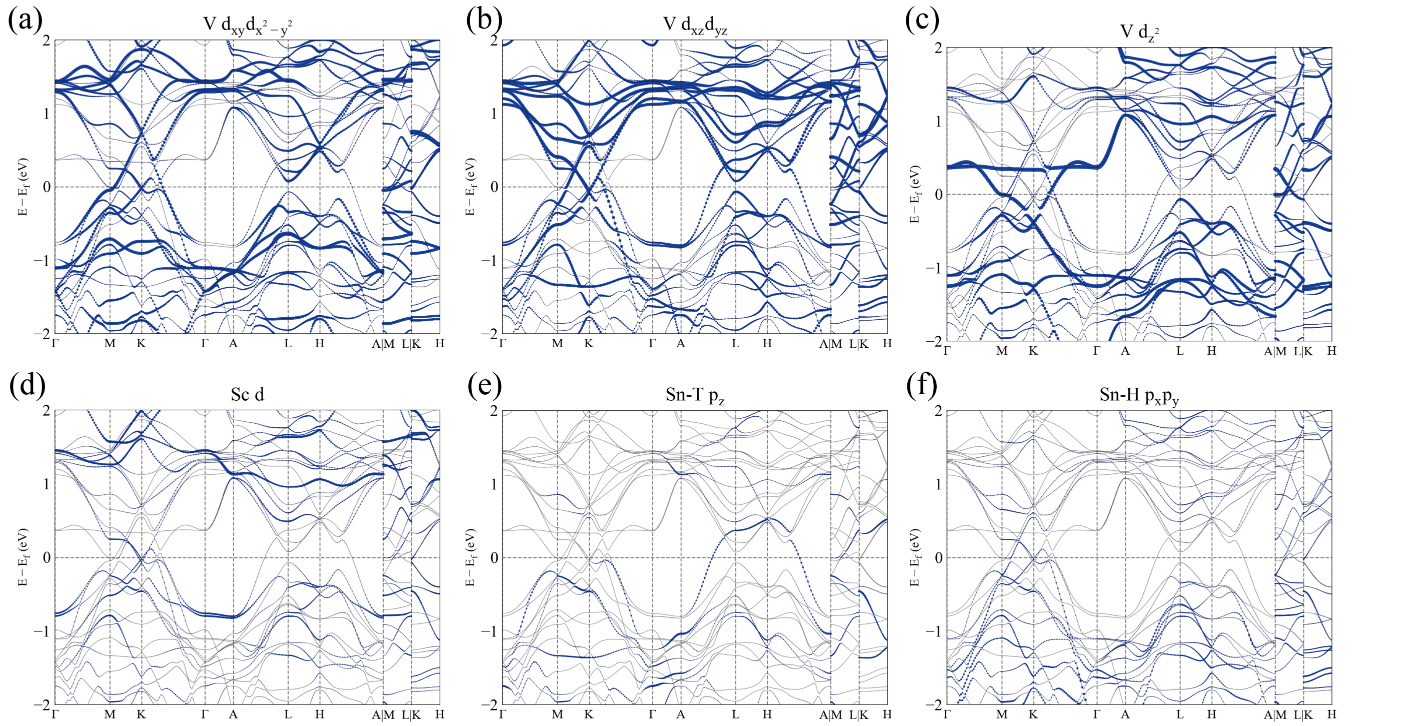


Supplementary Figure 23. The orbital projections of bands of $ScV_6Sn_6$ of the experimental pristine phase. (a) $d_{xy}, d_{x^2-y^2}$ orbitals of V. (b) $d_{xz}, d_{yz}$ of V. (c) $d_{z^2}$ of V. (d) All five $d$ orbitals of Sc. (e) $p_z$ of $Sn^T$. (f) $p_x, p_y$ of $Sn^H$.

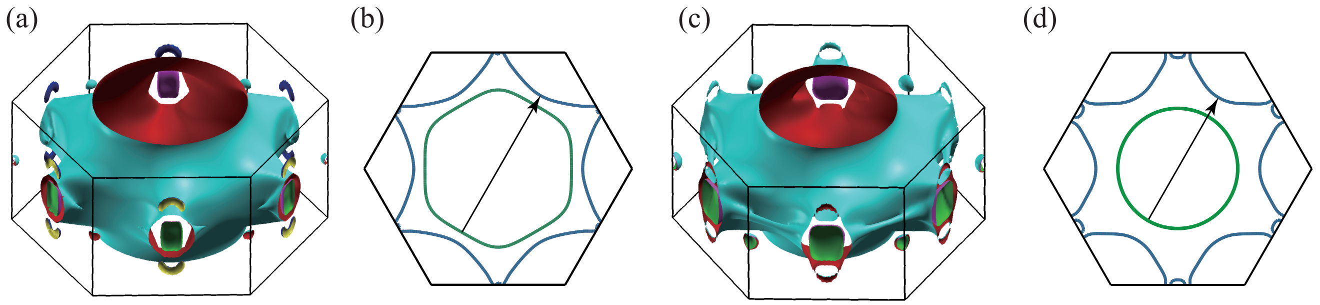


Supplementary Figure 24. The Fermi surfaces (FS) of pristine $ScV_6Sn_6$. (a) FS of the experimental structure[1]. (b) Two slices of FS, with the green line being $k_z = -0.32$ and the blue line being $k_z = 0.18$. The arrow denotes a weak FS nesting of $\boldsymbol{q}/(2\pi) = (0.4, 0.4, 0.5)$. (c) FS of the relaxed structure. (d) Two slices of FS, with the green line being $k_z/(2\pi) = 0.20$ and the blue line being $k_z/(2\pi) = 0.53$. The arrow denotes a weak FS nesting of $\boldsymbol{q}/(2\pi) = (\frac{1}{3}, \frac{1}{3}, \frac{1}{3})$.

two different slabs, one in which the top and the bottom surfaces are #4 and #1, respectively, and another in which the top and bottom surfaces are #2 and #3, respectively. A vacuum layer is inserted to separate the top and bottom surfaces, which only couple weakely in a large slab of four layers. For comparison purposes, in Supplementary Fig. 26, we also show the surface states' dispersion computed within a slab of 20 layers from the Wannier90 tight-binding (TB) model obtained from the bulk *ab-initio* band structure of $ScV_6Sn_6$[25]. A closer inspection reveals that the surface states computed using the two methods do show significant differences. This is because in Supplementary Fig. 26, the hopping amplitudes of the Wannier90 TB model are truncated abruptly at the surface, whereas in Supplementary Fig. 25, the hopping amplitudes near the surface are modified by the ground state charge distribution within the slab. As the *ab-initio* slab method used to compute the surface states in Supplementary Fig. 25 more accurately models the surface electronic charge distribution, we will use the results of Supplementary Fig. 25 for interpreting the ARPES results in what follows.

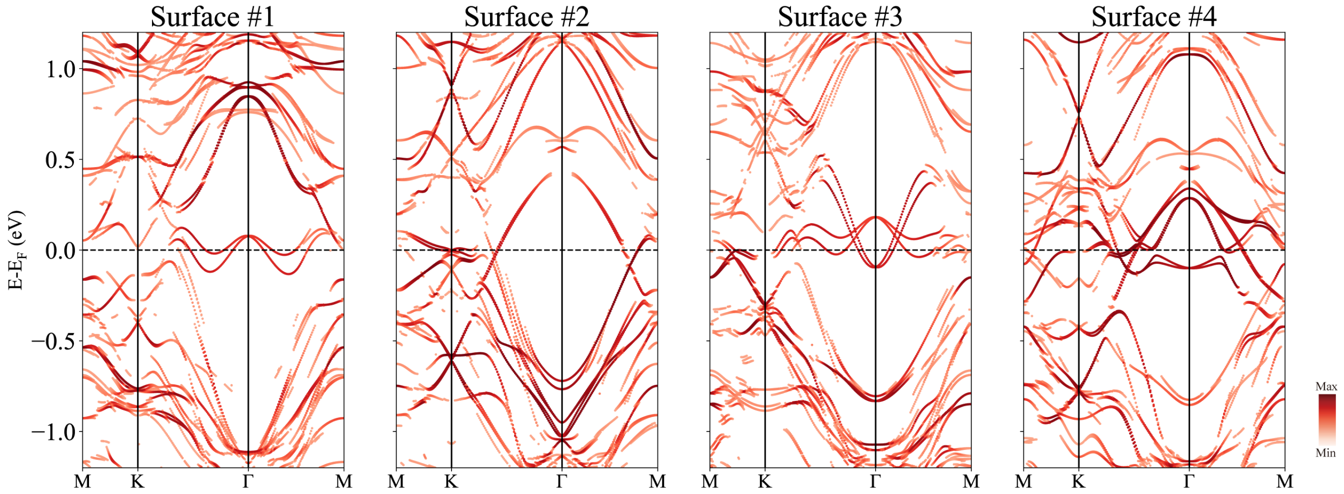


Supplementary Figure 25. The surface bands of $ScV_6Sn_6$ in the pristine phase, computed from a slab of 4 layers using VASP. The various surface terminations used are indicated above each panel, in the convention of Supplementary Fig. 1(a).

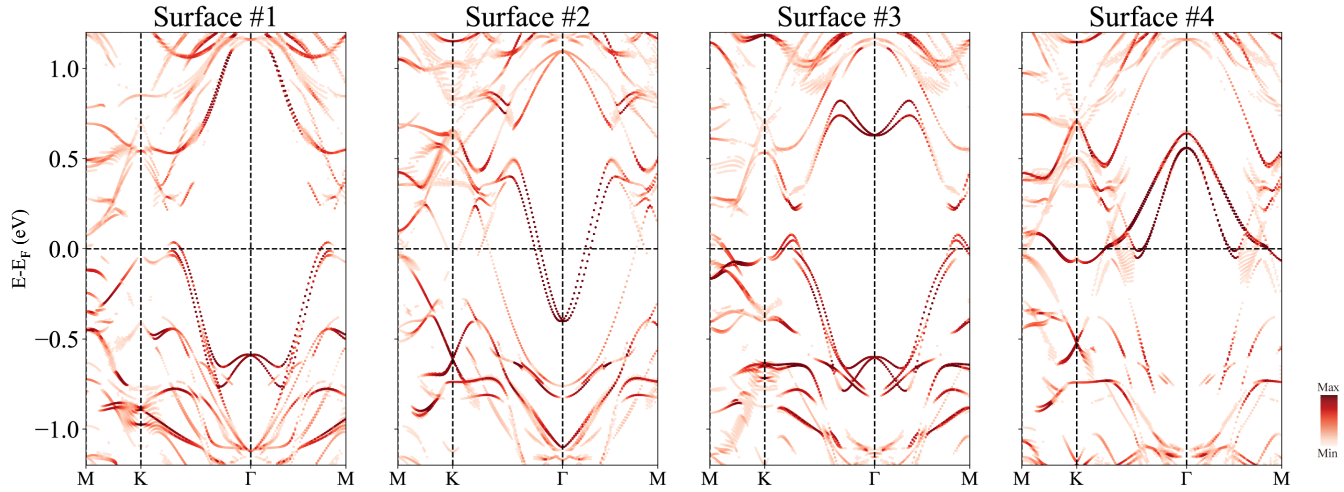


Supplementary Figure 26. The surface bands of $ScV_6Sn_6$ in the pristine phase, computed from a slab of 20 layers using a Wannier TB model of the *ab-initio* bulk bands from Supplementary Fig. 22(a). The various surface terminations used are indicated above each panel, in the convention of Supplementary Fig. 1(a).

### B. ARPES bands

In this section, we show the result of ARPES band structures in the CDW phase and their comparison with DFT results in Supplementary Fig. 28 and Supplementary Fig. 29 along two high symmetry directions: $\overline{\mathrm{M}}-\overline{\Gamma}-\overline{\mathrm{M}}$ and $\overline{\mathrm{K}}-\overline{\Gamma}-\overline{\mathrm{K}}$. In these figures, we show the band structure of ARPES superimposed the bulk bands of the non-CDW phase (black lines) and unfolded bulk bands of the CDW phase (red lines) from DFT.

Core-level spectra were taken before the ARPES experiments. In the 166 family, the kagome lattice can cleave with 4 terminations (Supplementary Fig. 1(a)). Two of them, $V_3Sn$ and ScSn, are energetically more favorable. Supplementary Fig. 27 summarizes the core level photoemission on pristine, freshly cleaved $ScV_6Sn_6$. Measuring the Sn 4*d* core level, we can resolve two types of terminations. Following the experimental observations of Y. Hu et al., [26], the ARPES data in the direction $\overline{\Gamma}-\overline{\mathrm{M}}$ was taken possibly in a $ScSn_2$ termination (sample #1). The direction $\overline{\mathrm{M}}-\overline{\mathrm{K}}-\overline{\Gamma}$ corresponds possibly to the $V_3Sn$ kagome

surface. The ARPES bands in Supplementary Fig. 28 are measured for sample #1 while Supplementary Fig. 29 are measured for sample #2. We summarize the main conclusions from the comparison of DFT and ARPES.

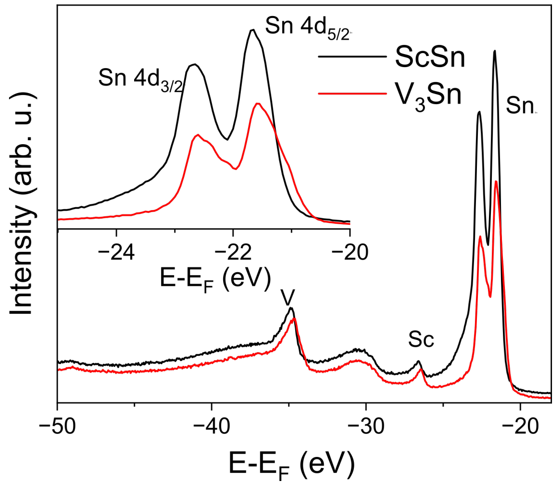


Supplementary Figure 27. Core level photoemission spectra on two different terminations. Inset, zoom in on the spin-orbit split Sn 4*d* states.

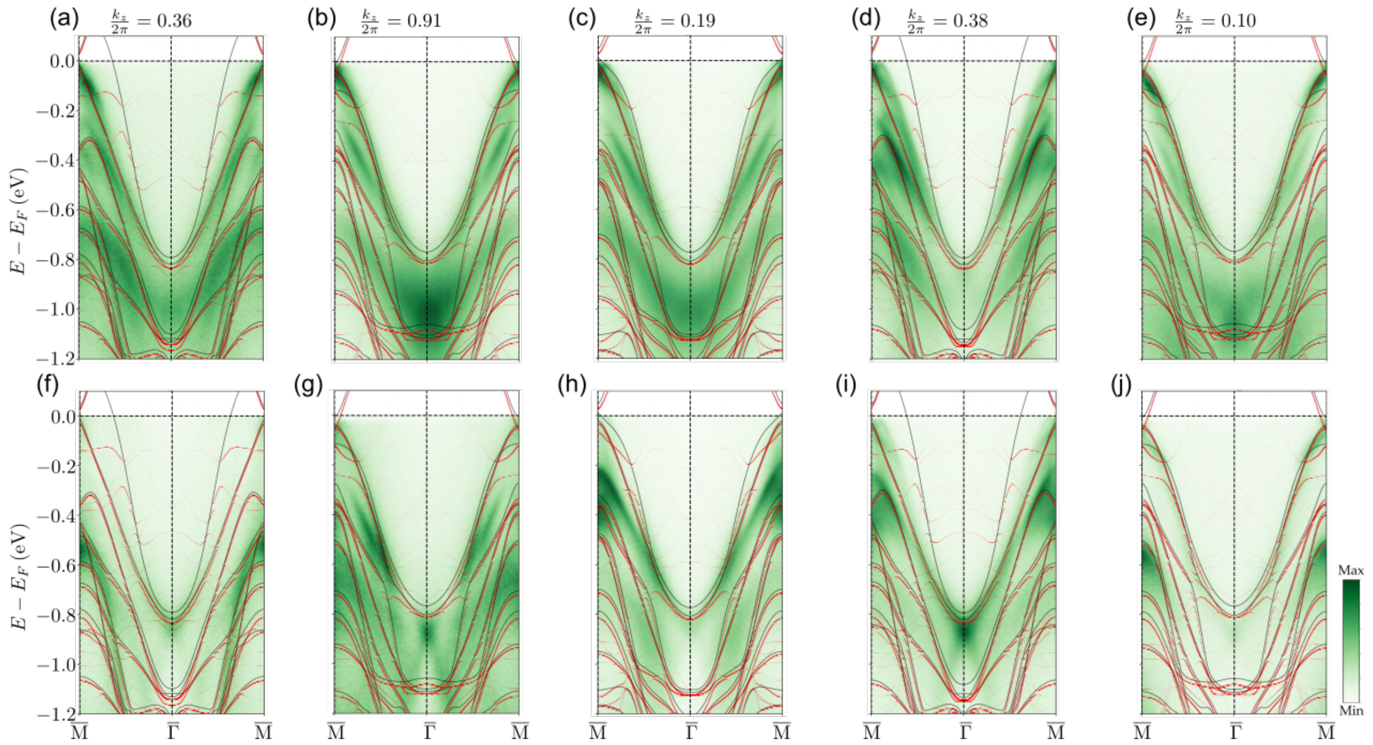


Supplementary Figure 28. The ARPES bands structures measured in the CDW phase at 7 K and comparison with DFT bands in $ScV_6Sn_6$ along $\overline{\mathrm{M}}-\overline{\Gamma}-\overline{\mathrm{M}}$ line. (a)-(e) ARPES Raw data measured with Linear Horizontal polarized light. The ARPES measurements are shown with superpositions of DFT, where the black lines are bulk bands of the pristine phase while the red lines are the unfolded bands of the CDW phase. (f)-(j) same as (a)-(e) but taken with Linear Vertical polarized light.

In Supplementary Fig. 28, we show the result along $\overline{\mathrm{M}}-\overline{\Gamma}-\overline{\mathrm{M}}$. The following conclusion can be drawn:

- There are good matches of the Fermi energy and Fermi velocities of the topmost occupied CDW bands on all $k_z$ slices.

- Close to the $k_z/2\pi = 0.5$ plane, i.e., $k_z/2\pi = 0.36$ and $0.38$ in Supplementary Fig. 28(a) and (d), the bands arising from the non-CDW phase (black lines) are absent in the ARPES data. This confirms the presence of the CDW. For the other

planes, the band structure of the CDW and non-CDW phases are quite similar. As a result, it is difficult to assess the presence of the CDW or normal phase in these $k_z$ slices.

- In all plots, there are always some bands as probed by ARPES which have the same Fermi velocities and have the same energy dispersions. These bands are present for the different $k_z$ and do not match with the DFT bulk bands. Therefore, these bands are surface states. These surface bands include:
  - One surface state with an energy of -0.25 eV at the $\overline{\mathrm{M}}$ point that comes down towards the $\Gamma$ point in Supplementary Fig. 28.
  - One surface state with an energy of -0.6 eV at the $\overline{\mathrm{M}}$ point that comes down towards the $\Gamma$ point in Supplementary Fig. 28.
  - One surface state with an energy of -1.1 eV at the $\overline{\Gamma}$ point appears in the ARPES data with L.H. polarization in Supplementary Fig. 28. The signal of this surface state vanishes with L.V. polarization due to the orbital character (Supplementary Fig. 28(f)-(j)).

  These surface states are confirmed from the DFT calculation with termination 2 (Kagome V bucked downward with Sn-T) in Supplementary Fig. 25.

- There is a suppression of the DOS at $\sim -0.20, -0.25$ eV due to the opening of small gaps in the CDW state, which is best seen at $k_z = 0.9$ and $k_z = 0.1$ in Supplementary Fig. 28(b)(e).

- The ARPES data with L.V. polarization shows an apparent crossing at approximately -0.85 eV at the Gamma point (which is not shown in DFT). However, this is not a true crossing. As the photoemission intensity is proportional to the matrix elements , this term vanishes due to the orbital character of these bands and the selection rules. As a result, there is a suppression of the spectral weight.

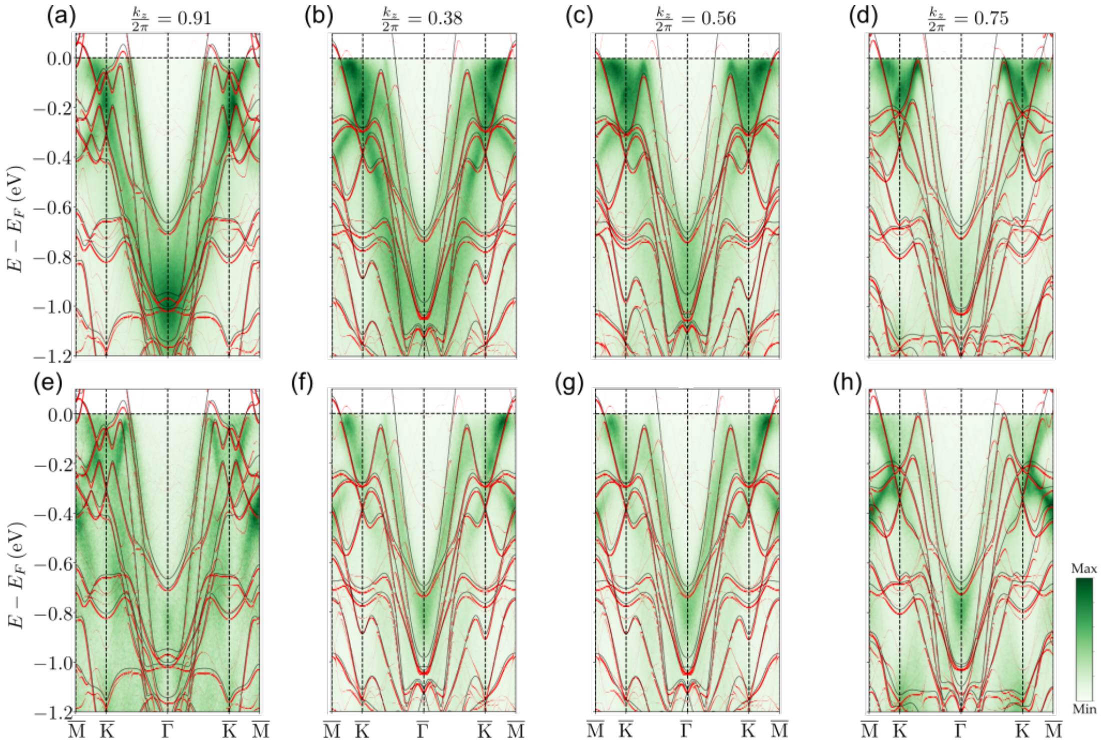


Supplementary Figure 29. The ARPES bands structures measured in the CDW phase at 7 K and comparison with DFT bands in $ScV_6Sn_6$ along $\overline{\mathrm{M}} - \overline{\mathrm{K}} - \overline{\Gamma} - \overline{\mathrm{K}} - \overline{\mathrm{M}}$ line. (a)-(d) ARPES Raw data measured with Linear Horizontal polarized light. The ARPES measurements are shown with superpositions of DFT, where the black lines are bulk bands of the pristine phase while the red lines are the unfolded bands of the CDW phase. DFT bands are shifted 100 meV upwards. (e)-(h) same as (a)-(d) but taken with Linear Vertical polarized light.

In Supplementary Fig. 29, we show the result along $\overline{\mathrm{M}} - \overline{\mathrm{K}} - \overline{\Gamma} - \overline{\mathrm{K}} - \overline{\mathrm{M}}$. The DFT bands match the ARPES bands well if they are shifted about 80-100 meV upwards. We compare the shifted DFT bands with ARPES:

- There are good matches of the Fermi energy and Fermi velocity of the topmost occupied CDW bands on all $k_z$ slices.
- There is little variation between the various $k_z$ planes. The energy of the top band changes, but its shape stays the same.
- There is little variation between the CDW and non-CDW DFT bands away from the $k_z = 0.5$ plane. Close to the $k_z = 0.5$ plane, the non-CDW bands that cross $E_F$ are not seen in the ARPES bands, confirming that they are gapped due to CDW.
- There is a very strong surface state appearing near the Fermi energy at the $\overline{\mathrm{K}}$ point. Sometimes this surface state is superimposed to an actual bulk state, for instance at $k_z = 0.91$ (Supplementary Fig. 29 (a)) . To make things more confusing, this surface state has the same shape as the top bulk band seen in DFT. The bulk DFT bands however shift in energy for different $k_z$ planes, whereas the surface state stays the same in all $k_z$ planes. At $k_z = 0.38$, the distance in energy between this surface state and the bulk band is larger, making easier to distinguish them (see Supplementary Fig. 29 (b)).
- For $k_z = 0.91$, there is a strong broad signal at the $\overline{\Gamma}$ point at about $-1.0$ eV in the L.H. ARPES signal (Supplementary Fig. 29 (a)). This has the same origin as in the $\overline{\mathrm{M}} - \overline{\Gamma} - \overline{\mathrm{M}}$ lines. In the L.V. ARPES plot, this gives rise to an apparent crossing.

[1] Arachchige, H. W. S. et al. Charge density wave in kagome lattice intermetallic $ScV_6Sn_6$. Physical Review Letters **129**, 216402 (2022).
[2] Hu, H. et al. To be published .
[3] Dyadkin, V., Pattison, P., Dmitriev, V. & Chernyshov, D. A new multipurpose diffractometer pilatus@snbl. Journal of Synchrotron Radiation **23**, 825–829 (2016).
[4] Rigaku. Rigaku Oxford Diffraction, CrysAlisPro Software system, Version 1.171.38.41 (2015).
[5] Sheldrick, G. M. *SHELXT* – Integrated space-group and crystal-structure determination. Acta Crystallographica Section A **71**, 3–8 (2015).
[6] Sheldrick, G. M. Crystal structure refinement with *SHELXL*. Acta Crystallographica Section C **71**, 3–8 (2015).
[7] Fåk, B. & Dorner, B. Phonon line shapes and excitation energies. Physica B: Condensed Matter **234-236**, 1107–1108 (1997). Proceedings of the First European Conference on Neutron Scattering.
[8] Diego, J. et al. van der waals driven anharmonic melting of the 3D charge density wave in VSe2. Nat. Commun. **12**, 598 (2021).
[9] Shirane, G., Shapiro, S. M. & Tranquada, J. M. Neutron Scattering with a Triple-Axis Spectrometer: Basic Techniques (Cambridge University Press, 2002).
[10] Kresse, G. & Hafner, J. Ab initio molecular-dynamics simulation of the liquid-metal–amorphous-semiconductor transition in germanium. Physical Review B **49**, 14251 (1994).
[11] Kresse, G. & Furthmüller, J. Efficient iterative schemes for ab initio total-energy calculations using a plane-wave basis set. Physical review B **54**, 11169 (1996).
[12] Kresse, G. & Furthmüller, J. Efficiency of ab-initio total energy calculations for metals and semiconductors using a plane-wave basis set. Computational materials science **6**, 15–50 (1996).
[13] Giannozzi, P. et al. Quantum espresso: a modular and open-source software project for quantum simulations of materials. Journal of physics: Condensed matter **21**, 395502 (2009).
[14] Perdew, J. P., Burke, K. & Ernzerhof, M. Generalized gradient approximation made simple. Physical review letters **77**, 3865 (1996).
[15] Togo, A. & Tanaka, I. First principles phonon calculations in materials science. Scripta Materialia **108**, 1–5 (2015).
[16] Prandini, G., Marrazzo, A., Castelli, I. E., Mounet, N. & Marzari, N. Precision and efficiency in solid-state pseudopotential calculations. npj Computational Materials **4**, 72 (2018).
[17] Yu, J. et al. Nontrivial quantum geometry and the strength of electron-phonon coupling. To be published .
[18] Cheng, S. et al. Nanoscale visualization and spectral fingerprints of the charge order in $ScV_6Sn_6$ distinct from other kagome metals. arXiv preprint arXiv:2302.12227 (2023).
[19] Kang, S.-H. et al. Emergence of a new band and the lifshitz transition in kagome metal $ScV_6Sn_6$ with charge density wave. arXiv preprint arXiv:2302.14041 (2023).
[20] Tuniz, M. et al. Dynamics and resilience of the charge density wave in a bilayer kagome metal. arXiv preprint arXiv:2302.10699 (2023).
[21] Hu, Y. et al. Phonon promoted charge density wave in topological kagome metal $ScV_6Sn_6$. arXiv preprint arXiv:2304.06431 (2023).
[22] Ku, W., Berlijn, T. & Lee, C.-C. Unfolding First-Principles Band Structures. Phys. Rev. Lett. **104**, 216401 (2010).
[23] Popescu, V. & Zunger, A. Extracting $e$ versus $\mathbf{k}$ effective band structure from supercell calculations on alloys and impurities. Phys. Rev. B **85**, 085201 (2012).
[24] Qijing, Z. QijingZheng/VaspBandUnfolding (2023).
[25] Wu, Q., Zhang, S., Song, H.-F., Troyer, M. & Soluyanov, A. A. WannierTools: An open-source software package for novel topological materials. Computer Physics Communications **224**, 405–416 (2018).
[26] Hu, Y. et al. Tunable topological dirac surface states and van hove singularities in kagome metal $GdV_6Sn_6$. Science Advances **8**, eadd2024 (2022).
